# Regime transitions in Arctic surface momentum balance reveal persistent and predictable dynamical states

**DOI:** 10.1038/s41467-026-75328-7

**Published:** 2026-07-08

**Authors:** Chao Liu, Xinfeng Liang

**Affiliations:** 1 Universit de Bretagne Occidentale, CNRS, Ifremer, IRD, Laboratoire d’Océanographie Physique et Spatiale (LOPS), IUEM, Plouzané, France; 2 School of Marine Science and Policy, University of Delaware, Lewes, DE USA

**Keywords:** Cryospheric science, Physical oceanography

## Abstract

Arctic sea-ice decline is fundamentally altering the momentum transfer between the atmosphere and ocean, with important implications for the climate system. However, capturing the complexity of air–ice–ocean interactions across varying timescales remains a challenge for climate models. Here, we show that Arctic Ekman pumping is organized into seven distinct physical regimes identified using a probabilistic clustering framework applied to a 26-year state estimate. These regimes reflect different combinations of wind, ice, and geostrophic forcing, alongside a residual component that becomes more prominent during seasonal sea-ice transitions. Spatially, the Beaufort Sea is characterized by frequent transitions and persistent ice–geostrophic coupled regimes, whereas the Nordic and Eurasian marginal seas exhibit greater variability in regime stability over time. This regime framework provides a physically grounded link between atmospheric forcing and surface momentum balance, offering a process-based perspective on how changing ice cover may influence Arctic upper-ocean dynamics.

## Abstract (web summary)

The study shows that Arctic Ekman pumping can be categorized into seven distinct regimes driven by varying wind, ice, and ocean forces, providing a process-based framework to understand how sea-ice decline alters atmosphere–ocean momentum transfer and upper ocean dynamics.

## Introduction

The Arctic has undergone profound sea-ice changes in recent decades as a consequence of Arctic amplification, with surface air temperatures rising at more than twice the global average rate^1,2^. The transition toward younger, thinner ice has reduced the mechanical strength of the ice cover, increasing its responsiveness to atmospheric forcing^3,4^. As a result, sea-ice drift speeds have accelerated, deformation has intensified, and ice motion has become increasingly sensitive to surface stress^5–7^. In parallel, the seasonal cycle of ice formation and melt has shifted, with earlier summer melt onset and delayed autumn freeze-up^8–10^. Observations indicate that ice advance has been delayed by more than a month since 1979^11^. These changes fundamentally alter air-ice-ocean coupling in the Arctic, particularly through surface stress and its role in driving Ekman transport and Ekman pumping^12,13^. Surface stress curl induces vertical exchange in the upper ocean, redistributing freshwater and heat beneath the ice and influencing stratification, circulation, and sea-ice evolution^14,15^. In the Beaufort Sea, persistent anticyclonic stress has been linked to enhanced Ekman convergence, deepening of the halocline, and long-term freshwater accumulation^16,17^. Ekman pumping in the Arctic has been examined using a wide range of observational, theoretical, and modeling approaches^18–23^. Studies that explicitly account for ice-ocean stress and ageostrophic dynamics demonstrate that ageostrophic currents and ice-ocean coupling can play a substantial role in Arctic momentum transfer^24–29^. At the same time, estimates of Ekman pumping remain highly sensitive to methodological choices, including how surface stress is decomposed and which current components are included^30,31^. Consequently, reported Ekman pumping magnitudes can vary by more than a factor of five even within the same region^32^. While the importance of various forcing mechanisms is well established, less attention has been paid to whether Arctic Ekman dynamics exhibit coherent large-scale structure when viewed through the relative balance of wind-, ice-, geostrophic-, and ageostrophic-driven forcing. Most previous studies emphasize regional case analyses, absolute magnitudes, or individual forcing terms^33,34^, making it difficult to assess how these mechanisms combine to form distinct dynamical states, how such states vary seasonally, or how they evolve on interannual timescales.

**Fig. 1:**
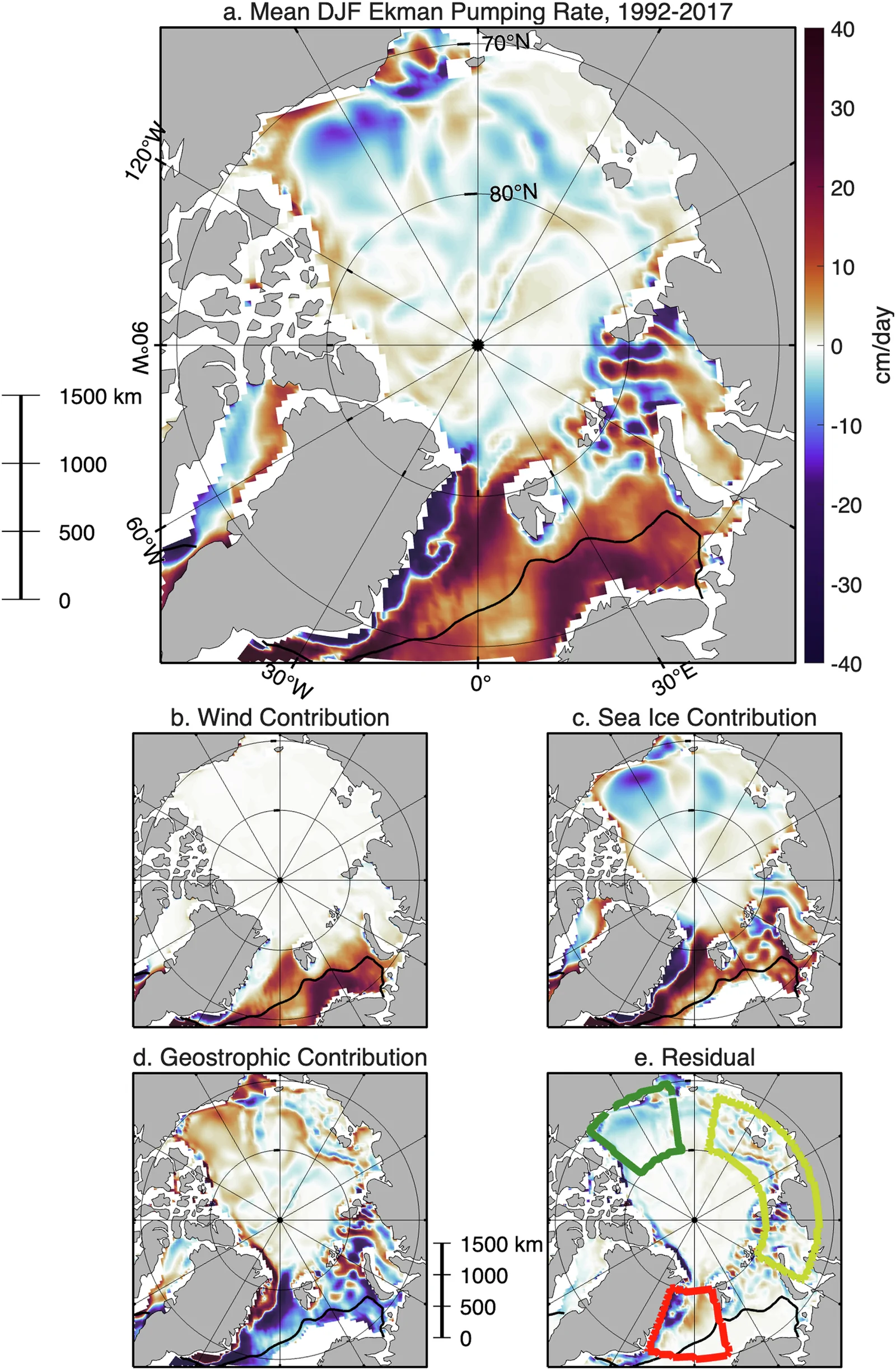
Seasonal climatology of Arctic Ekman pumping and its momentum-forcing components. Spatial distribution of (**a**) the mean winter (December to February) Ekman pumping rate in the Arctic Ocean over 1992–2017, and Ekman pumping associated with (**b**) wind forcing, (**c**) ice drift, (**d**) under-ice pumping caused by the geostrophic ocean current, and (**e**) a residual term representing contributions not captured by the explicitly resolved components. The black contour in each panel marks the median sea ice boundary; the green, red, and yellow boxes in Panel e define the Beaufort Gyre, Nordic Seas, and Eurasian regions. Positive values correspond to upward Ekman suction (upwelling), while negative values indicate downward Ekman pumping (downwelling).

In this work, we adopt a regime-based framework for Arctic Ekman dynamics, in which surface stress and Ekman pumping are characterized by the relative contributions of wind forcing, sea-ice motion, geostrophic currents, and a residual component (Fig. 1). Rather than focusing on individual processes in isolation, this framework provides a systematic way to organize the spatial, seasonal, and interannual variability of upper-ocean Ekman dynamics in terms of a small number of recurring forcing regimes. Using a dynamically consistent ocean-ice state estimate, we identify these regimes from seasonal climatologies with a probabilistic, unsupervised machine-learning approach. We then examine how the resulting Ekman forcing regimes are distributed across the Arctic, how their occurrence varies by season, and how they transition on interannual timescales, with particular focus on the Beaufort Gyre, Nordic Seas, and Eurasian regions. By construction, this approach offers an unbiased and physically interpretable synthesis of Arctic Ekman dynamics, and further provides a natural framework for assessing when satellite-based surface observations robustly capture upper-ocean Ekman processes and when additional, unresolved contributions become important.

## Results

### Spatial organization and physical robustness of Arctic Ekman pumping regimes

**Fig. 2:**
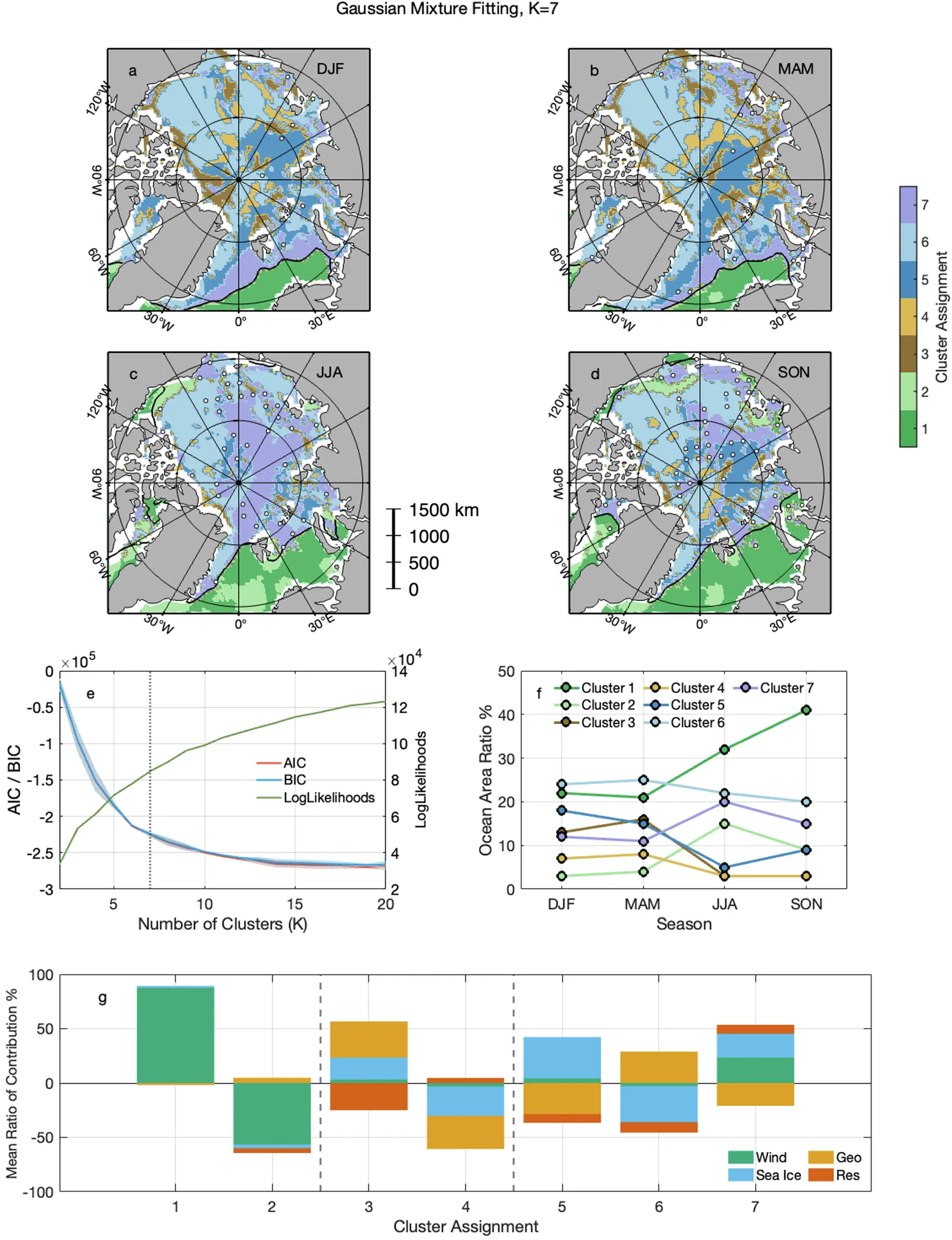
Machine-learning classification of Arctic Ekman pumping regimes. Gaussian Mixture Model clustering of seasonal Ekman pumping regimes. **a**–**d** Spatial distribution of the identified clusters (K = 7) in each season, based on the relative contributions of the four forcing terms. White dots indicate grid points where the maximum posterior cluster probability is below 0.7, reflecting low classification confidence. **e** Model selection metrics as a function of cluster number K, including the Akaike Information Criterion (AIC), Bayesian Information Criterion (BIC), and log-likelihood. Error bars denote ±2 standard deviations (2σ) estimated from 50 independent GMM realizations. **f** Seasonal area ratio occupied by each cluster across the Arctic Ocean. **g** mean contributions of each forcing term in each cluster.

Figure 2 presents the dynamic regimes of seasonal Ekman pumping identified by the GMM, reflected as varying organizations of distinct clusters. The first four panels (Fig. 2a–d) show the spatial distribution of the identified clusters (K = 7) in each season, based on the relative contributions of the four forcing terms, illustrating how regime structures reorganize throughout the year. Low-confidence classifications (probability <0.7) occur mainly in summer, consistent with enhanced dynamical variability and regime overlap. AIC and BIC (Fig. 2e) indicate diminishing improvement beyond K ≈ 7-10. The spatial coverage of each cluster across seasons (Fig. 2f) highlights their seasonal prevalence, and the mean Ekman pumping contributions for each forcing component within each cluster (Fig. 2g), which are computed by averaging the corresponding component values over all grid cells assigned to that cluster, provide a physical interpretation of the regimes in terms of dominant drivers.

The first 2 clusters (shown in green in Fig. 2a–d) represent regions that are almost exclusively wind-driven, corresponding to upwelling (C1) and downwelling (C2), respectively. Wind-driven upwelling occurs primarily in ice-free regions, particularly in the Greenland and Barents Seas. In contrast, wind-driven downwelling appears briefly during summer and autumn along the Beaufort Sea coast, with weaker signals also observed in the Laptev Sea. The area coverage of C1 exceeds 20% in winter and increases steadily to approximately 40% during the rest of the year. In comparison, C2 remains below 5% for most of the year, increases to about 14% in summer, and then decreases to roughly 10% in autumn.

Clusters C3 and C4 are characterized as sea-ice-geostrophic cooperative regimes, in which the ice and geostrophic contributions act in the same direction, with C3 and C4 differing primarily by a reversal in sign. This coherence indicates strong dynamical coupling between ice and ocean circulation, rather than purely resistive drag. C3 corresponds to an upwelling-favorable configuration, whereas C4 represents a downwelling regime. These two clusters occur primarily during winter and spring seasons, and are spatially scattered across the Arctic Basin. In terms of seasonal coverage, the upwelling regime (C3) occupies approximately 10-16% of the domain in winter and spring, before declining to below 5% during summer. In contrast, the downwelling regime (C4) rarely exceeds 10% coverage and exhibits comparable spatial extent to C3 during summer and autumn.

Clusters C5 and C6 represent the dominant dynamical regimes in the ice-covered Arctic, reflecting a balance between sea-ice-geostrophic forcings. In C5, ice drift induces Ekman upwelling, which is partially compensated by geostrophic downwelling, whereas C6 exhibits the opposite behavior, with geostrophic upwelling countered by ice-driven downwelling. Cluster C7 can be regarded as a sub-regime of C5, sharing the same sign structure (ice-driven upwelling and geostrophic downwelling) but distinguished by stronger wind-driven upwelling. Spatially, C5 and C7 occur primarily on the Eurasian side of the Arctic Basin, while C6 dominates the Amerasian Basin. This contrast likely reflects differences in large-scale circulation: the Eurasian sector is more strongly influenced by the transpolar drift and export-driven ice motion, whereas the Amerasian Basin is governed by the anticyclonic Beaufort Gyre, which enhances geostrophic control and suppresses ice-driven downwelling. Among these regimes, C6 occupies the largest area, covering approximately 25% of the domain in winter and decreasing to ~20% in summer. C5 remains below 20% year-round and declines to ~5% during summer. In contrast, C7 exhibits a pronounced seasonal increase, expanding from ~10% in winter to ~20% in summer, consistent with enhanced wind forcing and weakened ice resistance during the melt season.

### Regional differences and seasonal transitions of ice-ocean momentum transfer

We next focus on three dynamically active regions, the Beaufort Gyre, Nordic Seas, and Eurasian regions (Fig. 1e), all of which experience rapid changes in ocean circulation associated with evolving sea-ice conditions.

**Fig. 3:**
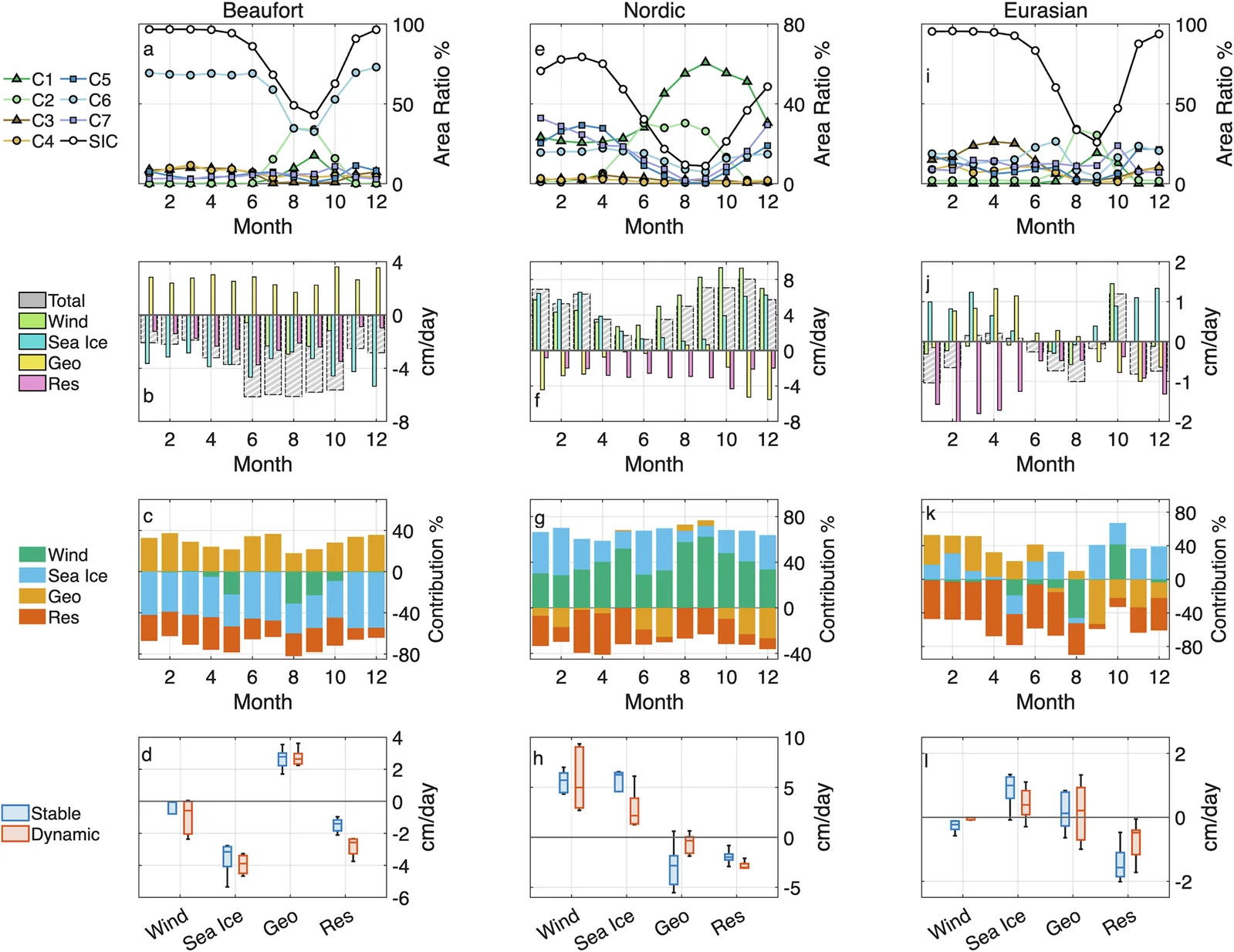
Seasonal variability of Arctic Ekman pumping regimes and forcing contributions in marginal sea regions. Decomposition of the Ekman pumping rate in the marginal sea regions marked in Fig. 1e. **a** Monthly area ratio occupied by each cluster in the Beaufort Gyre region. White dots mark the monthly sea ice concentration (SIC). **b** Seasonal cycle of Ekman pumping rate and its decomposed contributions in the region. **c** Same as (**b**) but as normalized mean contributions of each forcing term. **d** Mean Ekman pumping rate associated with each forcing term between the dynamic and stable months. For (**b**) & (**d**), positive values indicate upwelling, while negative values indicate downwelling. **e**–**h** Same as (**a**–**d**), but for the Nordic Seas region. **i**–**l** Same as (**a**–**d**), but for the Eurasian region.

In the Beaufort Gyre region (Fig. 3a), the dominant regime is C6, characterized by ice-drift-driven downwelling and geostrophic upwelling, resulting in a net downwelling state. This regime accounts for more than 70% of occurrences from November to June, but its spatial coverage decreases sharply to below 40% in August and September. This seasonal evolution is strongly correlated with the annual cycle of sea-ice concentration (*r* = 0.9, *p* < 0.05). In contrast, regime C2, associated with wind-driven downwelling, increases from nearly zero in winter to approximately 40% during late summer. At the same time, wind-driven upwelling briefly intensifies, reaching about 10% in August and 20% in September, particularly near the coast, consistent with enhanced coastal upwelling during ice-free conditions.

By directly examining the decomposed Ekman pumping components (Fig. 3b), we find that downwelling in the Beaufort Gyre region intensifies markedly from approximately −2 to −3 cm day⁻¹ in early summer to about −6 cm day⁻¹ between June and October. Although the sea-ice-drift contribution weakens moderately during summer (Fig. 3c), the dominant change arises from enhanced wind-driven downwelling, which increases to −3 to −4 cm day⁻¹, becoming comparable in magnitude to the ice-drift contribution.

Notably, the unresolved residual term is largest in June and October (Fig. 3c), corresponding to the seasonal melt and freeze-up periods. Here we categorize these months as “dynamic months,” defined by monthly sea-ice concentration changes exceeding 10%, in contrast to more “stable months.” Student-t testing shows that the residual term is the only component exhibiting a significant increase during dynamic months (Fig. 3d). As the Arctic is facing a longer melt season and a shorter freeze season^9^, this implies intensified transient momentum changes during rapid sea ice evolution.

In the Nordic Seas (Fig. 3e), seasonal sea-ice concentration is substantially lower than in the Beaufort Gyre region, ranging from a winter maximum of about 70% to summer minima near 10% in August and September. As a result, ice-drift- and geostrophic-dominated regimes (i.e., C3-C6) are generally not prevalent, occurring only briefly from January to April. From May to September, the coverage of regime C1 nearly doubles, increasing from ~32% to ~65%, indicating a transition toward dominant wind-driven upwelling.

Consistent with this pattern, the decomposition of Ekman upwelling shows that the wind-driven component exhibits a pronounced seasonal cycle that closely mirrors the seasonal variation of C1 (Fig. 3f). Wind-driven upwelling reaches a minimum of approximately 3 cm day⁻¹ in May-June and intensifies to more than 9 cm day⁻¹ in October-November. Contributions from sea-ice drift and geostrophic flow also follow the seasonal evolution of regimes C5-C7 and are strongly correlated with sea-ice concentration. The downwelling associated with geostrophic currents peaks later in the year, exceeding −4 cm day⁻¹ in November–December.

Notably, the residual term remains relatively stable between April and October, with values between −3 and −4 cm day⁻¹, but weakens during winter (December–March) to less than −2 cm day⁻¹. Owing to this stability, the relative contribution of the residual term is particularly high from March to May and from August to October (Fig. 3h).

In the Eurasian sector (Fig. 3i), no single regime dominates throughout the year, reflecting a more spatially heterogeneous and dynamically mixed environment compared to the Beaufort Gyre and Nordic Seas. Regime C3 is most frequent in spring, transitioning to C2 in summer, while autumn and winter are primarily characterized by C6 and episodically C7. Notably, regime occurrence generally remains below ~30%, except for C2 in summer, which approaches ~40%, indicating weaker regime dominance and more distributed forcing conditions.

The corresponding Ekman pumping components (Fig. 3j) reveal a clear seasonal structure in the geostrophic contribution, which transitions from positive values in spring to negative values in autumn, with amplitudes of approximately ±1 cm day⁻¹. In contrast, the sea-ice drift contribution remains predominantly positive throughout most of the year, except during summer when reduced ice concentration weakens its influence. The wind-driven component is relatively weak (generally <0.5 cm day⁻¹) but exhibits a pronounced peak in October ( ~ 1.4 cm day⁻¹), consistent with enhanced air-sea momentum transfer under reduced ice cover.

The residual term remains substantial across all seasons, particularly during winter and spring, with magnitudes ranging from −1 to −2 cm day⁻¹ and accounting for up to ~40% of the total Ekman pumping (Fig. 3k). This persistent and comparatively large residual highlights a significant portion of the momentum balance not captured by wind, ice-drift, or geostrophic contributions. Notably, only the residual term exhibits a significant change during dynamical months across all three examined regions.

### Contrasting stability and interannual regime transitions

Here, we focus on wintertime transitions of the dynamical regimes, as this period is characterized by relatively stable surface conditions and reduced complexity from melt-related freshwater inputs. This allows wind- and ice-driven momentum transfer to be more clearly interpreted in the Ekman response, despite the presence of buoyancy forcing associated with brine rejection during sea-ice formation.

We first examine how surface Ekman regimes transition over the full 26-year record. A transition matrix P is constructed by counting area-weighted transitions of each grid cell between cluster regimes that change state from one year to the next, and normalizing each row to unity. The element ${{{\rm{P}}}}_{{{\rm{ij}}}}$, therefore, represents the probability that a grid cell in regime i at time t transitions to regime j at time t + 1, with diagonal elements quantifying regime persistence.

**Fig. 4:**
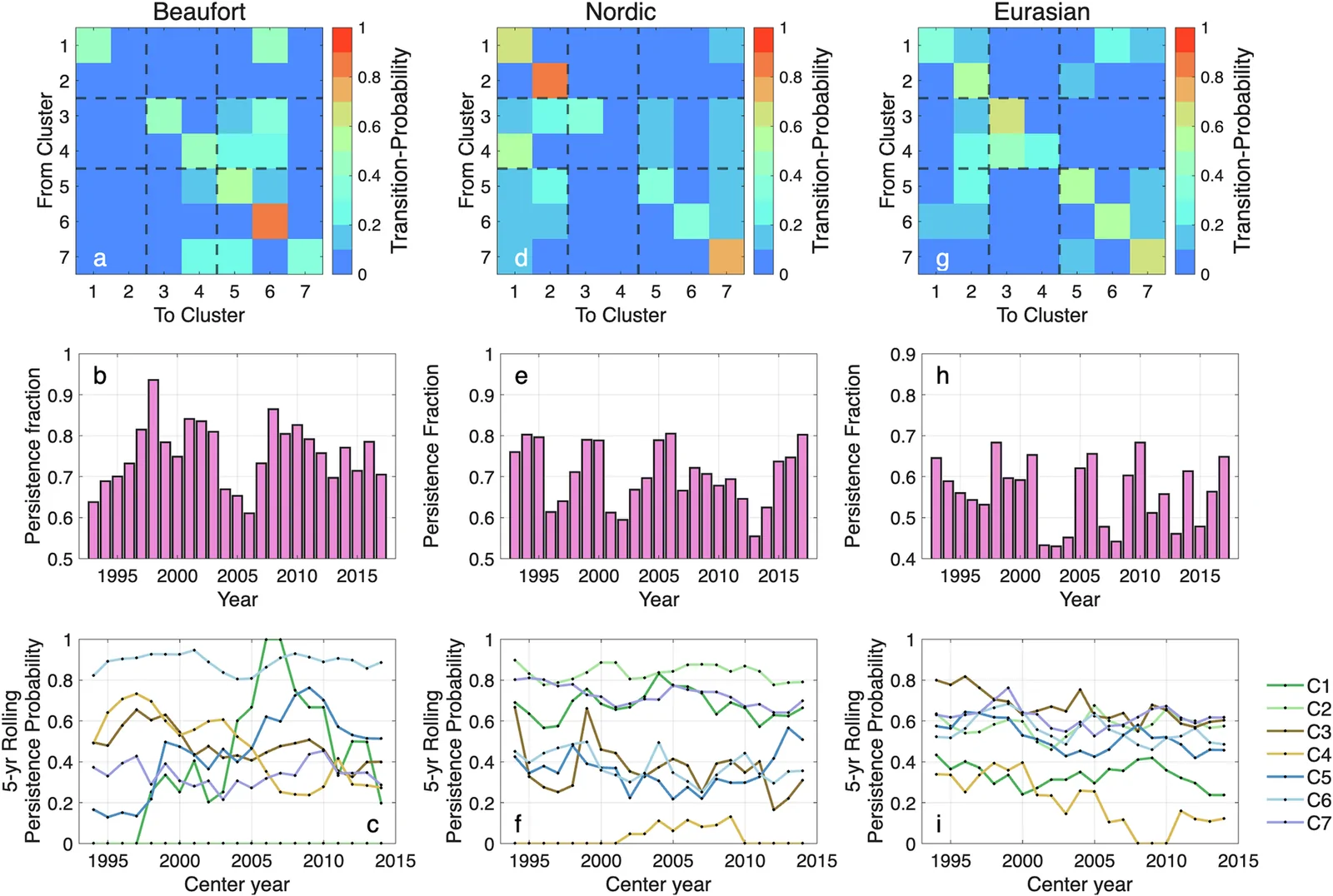
Interannual transitions and persistence of Arctic Ekman pumping regimes. Interannual transitions of dynamical regimes in the marginal sea regions during winter (DJF, 1992-2017). **a** Area-weighted transition probability matrix between clusters for the Beaufort Gyre region. **b** Annual persistence, defined as the fraction of area where the assigned cluster remains unchanged relative to the previous winter. **c** Five-year rolling persistence, defined as the probability that a cluster remains unchanged from the previous winter. **d**–**f** Same as (**a**–**c**), but for the Nordic Seas region. **g**–**i** Same as (**a**–**c**), but for the Eurasian region.

In the Beaufort Gyre region (Fig. 4a), the off-diagonal elements (${{{\rm{P}}}}_{{{\rm{ij}}}}$) are interpreted as preferred pathways of state change, representing the conditional probabilities of transitions among clusters. The dominant transitions occur from C3-C4 toward C5-C6, indicating that these intermediate ice-geostrophic regimes act as attracting transition states in phase space, while weaker reverse transitions from C5 and C7 back toward C4 are also present.

In contrast, persistence diagnostics (Fig. 4b–c) describe the probability of remaining within the same regime (i.e., the diagonal elements of the transition matrix, ${{{\rm{P}}}}_{{{\rm{ii}}}}$) and its temporal variability, and are therefore complementary to the transition probabilities shown in Fig. 4a. In the Beaufort Gyre region, approximately 70% of the area remains in the same regime from one winter to the next (Fig. 4b), indicating generally stable stress-Ekman coupling. This fraction decreases to ~60% during 2004–2006, followed by a rebound to ~80% in subsequent years.

At the regime level (Fig. 4c), C6 exhibits the highest persistence, remaining between 0.8 and 0.9 throughout most of the record. In contrast, C1 shows pronounced non-monotonic variability, increasing from ~0.2 in 2002 to nearly unity in 2007–2008 before declining thereafter. C5 shows a moderate increase in persistence probability during the early-to-mid period (from ~0.4 to ~0.8), followed by relative stabilization. Conversely, C3 and C4 show gradual reductions in persistence from values above ~0.6 to approximately ~0.3–0.4 over the record.

In the Nordic Seas (Fig. 4d), transitions from C3-C7 toward C1-C2 dominate, while in the Eurasian Basin (Fig. 4g), transitions toward C2 are most common, although with generally weaker probabilities. These patterns suggest that ice-geostrophic cooperative regimes function as intermediate dynamical states connecting wind-dominated and more balanced configurations.

In the Nordic Seas, interannual variability in total persistence ranges from approximately 50–60% at its lowest to nearly 80% at its highest (Fig. 4e). At the individual regime level (Fig. 4f), C1, C2, and C7 show gradual reductions in persistence toward the end of the record, while C5 and C6 exhibit relatively higher persistence values during later periods. C3 displays pronounced variability prior to 2000, ranging between ~0.6 and ~0.2.

In the Eurasian Basin, persistence variability is lower than in the other two regions, ranging from just above 40% to less than 70%, with values below 50% for roughly half of the period after 2000 (Fig. 4h), indicating comparatively weaker regime stability. At the cluster level (Fig. 4i), most regimes exhibit gradual but modest temporal variability in persistence. In particular, C3 decreases from ~0.8 to ~0.6, while C1 and C4 show reductions of approximately ~0.2, whereas the remaining clusters remain broadly stable.

## Discussion

By reframing Arctic surface momentum balance in terms of dynamically coherent regimes, this study moves beyond regional diagnostics toward a basin-scale, process-oriented interpretation. The clustering reveals a small number of recurring momentum balance states that are physically interpretable and persistent, linking wind-driven, ice-ocean cooperative, and ice-ocean balanced regimes across seasons and regions. This regime structure provides a unifying framework that reconciles previously disparate regional analyses^32,35^ within a consistent Arctic-wide context.

The regime framework also clarifies the apparent discrepancy between regional extremes and basin-scale statistics. Regions such as the Beaufort Gyre region and Nordic Seas do not necessarily stand out in relative-contribution clustering because their distinctive behavior lies primarily in the magnitude of momentum terms rather than their fractional partitioning. Once interpreted through the lens of regime transitions and persistence, however, as shown in Fig. 4a–b, these regions exhibit distinct dynamical signatures: the Beaufort Gyre region is characterized by high regime stability and gradual transitions toward ice-ocean balanced states, whereas the Nordic Seas displays frequent transitions and lower persistence, consistent with its role as a dynamically forced export gateway.

The transition probability analysis further highlights strong contrasts in temporal organization. The Beaufort Gyre region exhibits high interannual persistence, with approximately 70-80% of the area remaining in the same regime from year to year, implying a degree of dynamical memory and enhanced predictability. In contrast, the Nordic Seas show substantially higher variability, reflecting rapid regime shifts driven by external forcing and advection. These differences emphasize that Arctic momentum balance predictability is strongly region- and regime-dependent, rather than uniform across the basin.

The regime-based perspective connects physical interpretation with observational capability and predictability. The non-residual momentum contributors, including surface wind stress, sea-ice motion, and geostrophic currents, are constrained by satellite observations to varying degrees^36–40^. Sea-ice drift and sea surface height are directly observed, whereas surface wind stress is typically derived from atmospheric reanalyzes and parameterized air-ice-ocean drag formulations. As a result, the accuracy of these terms varies spatially, with larger uncertainties in regions of high sea-ice concentration. Despite these limitations, the dominant contributors identified here remain tied to quantities that are routinely monitored at the pan-Arctic scale^41–45^.

Another important result of this study is that large residual contributions emerge as a defining property of specific regimes, distinguishing them from those dominated by resolved wind, ice-drift, and geostrophic components. In the ECCO framework, this residual reflects unresolved or parameterized processes, including possible candidates such as high-frequency ice-ocean coupling variability, temporal averaging effects, and model-observation mismatches. While analogous residuals in the real Arctic may involve processes such as internal ice stress divergence, small-scale deformation, and variable ice-ocean drag, these mechanisms are not explicitly represented in ECCO and should therefore not be directly inferred. Rather than being treated as noise, the residual term provides a physically meaningful diagnostic of where present observing systems and model formulations remain insufficient^46^. The spatial coherence and temporal persistence of these residual-dominated regimes further support their physical relevance. As such, this framework offers a pathway for evaluating coupled ice-ocean models using regime statistics and transitions, providing physically grounded benchmarks that are directly tied to current and future Arctic observing capabilities.

While the present analysis focuses on surface momentum balance, the identified regimes provide a physically interpretable framework for examining how different forcing configurations may influence upper-ocean structure. Preliminary results indicate systematic differences in mixed-layer depth across regimes (Fig. S5), suggesting that similar surface forcing can be associated with distinct upper-ocean responses depending on the dynamical state. However, the translation of surface stress into vertical exchange is ultimately modulated by stratification, which is not explicitly resolved in this framework. We therefore interpret the regime transitions primarily as changes in the forcing environment of vertical exchange rather than direct predictors of mixing intensity. This distinction is consistent with observational evidence that stratification can strongly limit the penetration of turbulence into the ocean interior, while weaker stratification allows wind-driven mixing to extend to intermediate depths in ice-free Arctic conditions^47,48^.

## Methods

### The ECCO version 4 ocean state estimate

We use Version 4, Release 4 of the Estimating the Circulation and Climate of the Ocean state estimate^49,50^ (ECCOv4r4), produced with the Massachusetts Institute of Technology General Circulation Model^51^ (MITgcm) coupled to a dynamic-thermodynamic sea-ice model^52^. The solution spans 1992–2017 and is dynamically consistent, satisfying conservation of mass, momentum, and energy while assimilating a broad range of satellite and in situ observations.

The monthly ECCOv4r4 fields are provided on the LLC90 grid, with horizontal resolution of ~22–40 km in the Arctic and 50 vertical levels with enhanced upper-ocean resolution. Surface momentum forcing is derived from 6-hourly ERA-Interim reanalysis^53^ using bulk formulation^54^. The assimilation includes satellite altimetry, bottom pressure, Argo floats, Ice-Tethered Profilers, and mooring observations, enabling physically consistent air-ice-ocean coupling.

ECCOv4r4 has been widely used to diagnose Arctic circulation and upper-ocean processes, including Ekman transport and pumping^55–57^, making it well suited for the regime-based analysis presented here. While systematic differences in magnitude between ECCO and observation-based estimates remain (Figs. S1–S4, Table S1), we emphasize that the present analysis focuses on relative contributions and regime structure, which are governed by variability and co-variability across components rather than absolute magnitudes. Because the identified regimes are derived from ECCOv4r4 surface momentum fields, biases relative to observation-based estimates may influence not only the temporal variability of the diagnosed momentum components, but also the spatial distribution and relative occurrence of the identified regimes.

### Ekman pumping estimates

Ekman dynamics in the upper Arctic Ocean have been extensively investigated using observational, theoretical, and modeling approaches. The Ekman upwelling (or pumping) rate is commonly expressed as

1 $${w}_{e}=\frac{1}{f{\rho }_{w}}{\nabla \times {{\boldsymbol{\tau }}}}_{{{\boldsymbol{o}}}}$$

where ${\tau }_{o}$ is the ocean-surface stress, f is the Coriolis parameter, and ${\rho }_{w}$ is the water density.

Adopting the framework developed by Meneghello et al.^58^, ${w}_{e}$ and can be decomposed into contributions associated with different surface forcings, including terms associated with air-sea wind stress (${w}_{a}$) and ice-ocean stress (${w}_{i}$), a term associated with geostrophic surface currents (${w}_{g}$), and a residual term (${w}_{r}$) representing contributions not captured by the explicitly resolved components (Fig. 1). This decomposition enables a quantitative attribution of Ekman pumping to distinct dynamical processes under varying ice and circulation conditions^32,33,35,59^. Detailed calculation is provided in supplementary.

### Gaussian mixture modeling

Gaussian Mixture Modeling (GMM) is used to identify distinct regimes in a four-dimensional feature space defined by the normalized ratios (γ) of the climatological mean contributions of the four Ekman pumping components as shown in Fig. 1. For term x, there is:

2 $${\gamma }_{x}=\frac{{w}_{x}}{{{|w}}_{a}|+|{w}_{i}|+{{|w}}_{g}|+{{|w}}_{r}|}$$

where the sign of each component is retained separately to distinguish upwelling and downwelling regimes. Noting the input data contains on spatial or temporal coordinates to ensure unbiased results.

GMM represents the data as a mixture of multivariate Gaussian distributions, allowing clusters to differ in shape, orientation, and overlap^60^. Compared to K-means clustering, which assumes spherical clusters and hard assignments^61^, GMM provides greater flexibility and a probabilistic classification, enabling a more nuanced representation of regime boundaries^62^.

To ensure robustness, the number of clusters is selected using 5-fold cross-validation combined with penalized likelihood criteria, including the Akaike Information Criterion (AIC) and Bayesian Information Criterion (BIC). These metrics balance model fit against complexity, reducing the risk of overfitting and guiding the selection of an optimal and interpretable number of regimes.

## Supplementary information

Supplementary Information

Transparent Peer Review file

## Data Availability

The ECCO-V4r4 output is distributed by the Physical Oceanography Distributed Active Archive Center (PO.DAAC, https://podaac.jpl.nasa.gov/ECCO/). Additionally, following data are used for the supplementary material: Sea ice data are freely accessible at NSIDC (ice motion: 10.5067/INAWUWO7QH7B; ice extent: 10.5067/MPYG15WAA4WX). Arctic sea level is provided at AVISO. The surface stress fields are available at 10.5281/zenodo.15534576.

## Code availability

The scripts for this study are available at 10.5281/zenodo.19519590. The analysis of ECCO-V4r4 was performed with the gcmfaces toolbox freely available from https://gcmfaces.readthedocs.io/en/latest/.
