# Supplementary material for: Regime transitions in Arctic surface momentum balance reveal persistent and predictable dynamical states
Source: Nat Commun. 2026 Jul 8;17:9132. doi: 10.1038/s41467-026-75328-7 (PMC13518975; doi:10.1038/s41467-026-75328-7)
Supplement: Supplementary file 1 [file 41467_2026_75328_MOESM1_ESM.pdf]

Supplementary Information for

Regime Transitions in Arctic Surface Momentum Balance Reveal Persistent and Predictable Dynamical States

Chao Liu[1], Xinfeng Liang[2]

1 Universit de Bretagne Occidentale, CNRS, Ifremer, IRD, Laboratoire d'Océanographie Physique et Spatiale (LOPS), IUEM, Plouzané, France

2 School of Marine Science and Policy, University of Delaware, Lewes, DE, USA

Ekman Pumping Estimates

The Ekman pumping rate is computed as

$$w_e = \frac{1}{f\rho_w} \nabla \times \boldsymbol{\tau}_o$$

where f is the Coriolis parameter and $\rho_w = 1027.5 \text{ kg m}^{-3}$ is seawater density. Following Meneghello et al. (2017, 2018), the total surface stress $\boldsymbol{\tau}_o$ is expressed as a weighted combination of ice–ocean and air–ocean stresses:

$$\boldsymbol{\tau}_o = \alpha\boldsymbol{\tau}_i + (1-\alpha)\boldsymbol{\tau}_a \qquad \text{(S1)}$$

where α is sea-ice concentration, $\boldsymbol{\tau}_a$ is wind stress, and $\boldsymbol{\tau}_i$ is ice–ocean stress.

The wind-driven Ekman pumping contribution is therefore

$$w_a = \frac{1}{f\rho_w} \nabla \times [(1-\alpha)\boldsymbol{\tau}_a] \qquad \text{(S2)}$$

The ice–ocean stress is computed using a quadratic drag law $\boldsymbol{\tau}_i = \rho_w C_D |\mathbf{U}_{rel}|\mathbf{U}_{rel}$, where $C_D = 0.0055$ is the drag coefficient and $\mathbf{U}_{rel}$ is the relative velocity between ice and ocean surface currents.

The relative velocity is approximated as $\mathbf{U}_{rel} = \mathbf{U}_i - (\mathbf{U}_e + \mathbf{U}_g)$ where $\mathbf{U}_i$ is ice drift velocity, $\mathbf{U}_g$ is geostrophic velocity derived from dynamic ocean topography, and $\mathbf{U}_e$is the Ekman velocity.

To isolate the ice-drift contribution, geostrophic velocity is set to zero when computing $\mathbf{U}_{rel}$, yielding an ice-only stress $\boldsymbol{\tau}_{i0}$. The corresponding Ekman pumping is

$$w_i = \frac{1}{f\rho_w} \nabla \times [\alpha\boldsymbol{\tau}_{i0}] \qquad \text{(S3)}$$

The ice–ocean stress associated with geostrophic currents is defined as

$$\boldsymbol{\tau}_g = \boldsymbol{\tau}_i - \boldsymbol{\tau}_{i0}$$

with the corresponding Ekman pumping

$$w_g = \frac{1}{f\rho_w} \nabla \times [\alpha\boldsymbol{\tau}_g] \qquad \text{(S4)}$$

Using the full surface velocity from ECCOv4r4 instead of $\mathbf{U}_e + \mathbf{U}_g$, which satisfies volume conservation, we also compute a fully resolved ice–ocean stress $\boldsymbol{\tau}_{if}$ and associated pumping w_{if}. A residual term representing contributions not explicitly resolved is defined as

$$w_r = w_{if} - (w_i + w_g) \qquad \text{(S5)}$$

Comparison with Satellite Dataset

For comparison, we also utilized the following satellite-based input datasets to calculate the total ocean-surface stress (Eqs. S1–S5): ocean-surface wind stress (τ_{aw}), sea-ice concentration (α), sea-ice motion (U_{ice}), and dynamic topography for geostrophic velocity (U_g).

Surface stress fields (both air–water and ice–ocean stress) are obtained from Liu & Yu (2025), provided as a daily 25 km EASE-Grid product spanning 2011–2021. It is calculated using a bulk parameterization approach that combines ocean-surface winds, ice motion vectors, and sea surface height data from multiple satellite platforms. The air-sea stress is derived using the Coupled Ocean–Atmosphere Response Experiment (COARE) bulk flux algorithm (version 3.6; COARE bulk flux algorithm version 3.6).

Daily sea-ice motion vectors are obtained from the National Snow and Ice Data Center Polar Pathfinder Daily 25 km EASE-Grid Sea-Ice Motion Vectors (version 4; NSIDC Polar Pathfinder Sea Ice Motion Vectors version 4), covering 1978–2023. This product integrates multiple satellite and in situ observations, including passive microwave radiometers (SSM/I, AMSR-E), infrared and visible sensors (AVHRR, MODIS), scatterometers (QuikSCAT), drifting buoys, and atmospheric reanalysis.

Sea-ice concentration is obtained from the Nimbus-7 SMMR and DMSP SSM/I-SSMIS Passive Microwave Data version 2 (NSIDC-0051), which provides a continuous, long-term record since 1987 on a 25 km polar stereographic grid.

Geostrophic surface velocity is derived from the multi-altimeter sea-level product developed by CLS/PML (Prandi et al., 2021). This dataset provides sea surface height and derived geostrophic currents north of 50° N on a 25 km EASE2 grid, with an effective temporal resolution of approximately 3 days.

The surface stress fields are available at https://doi.org/10.5281/zenodo.15534576 (Liu and Yu, 2024). Sea ice data are hosted by NSIDC (ice motion: https://doi.org/10.5067/INAWUWO7QH7B, Tschudi et al., 2019; ice extent: https://doi.org/10.5067/MPYG15WAA4WX, DiGirolamo et al., 2022), and sea level data by AVISO (dynamic topography:

https://www.aviso.altimetry.fr/en/data/products/sea-surface-height-products/regional/arctic-ocean-sea-level-heights.html, Prandi et al., 2021).

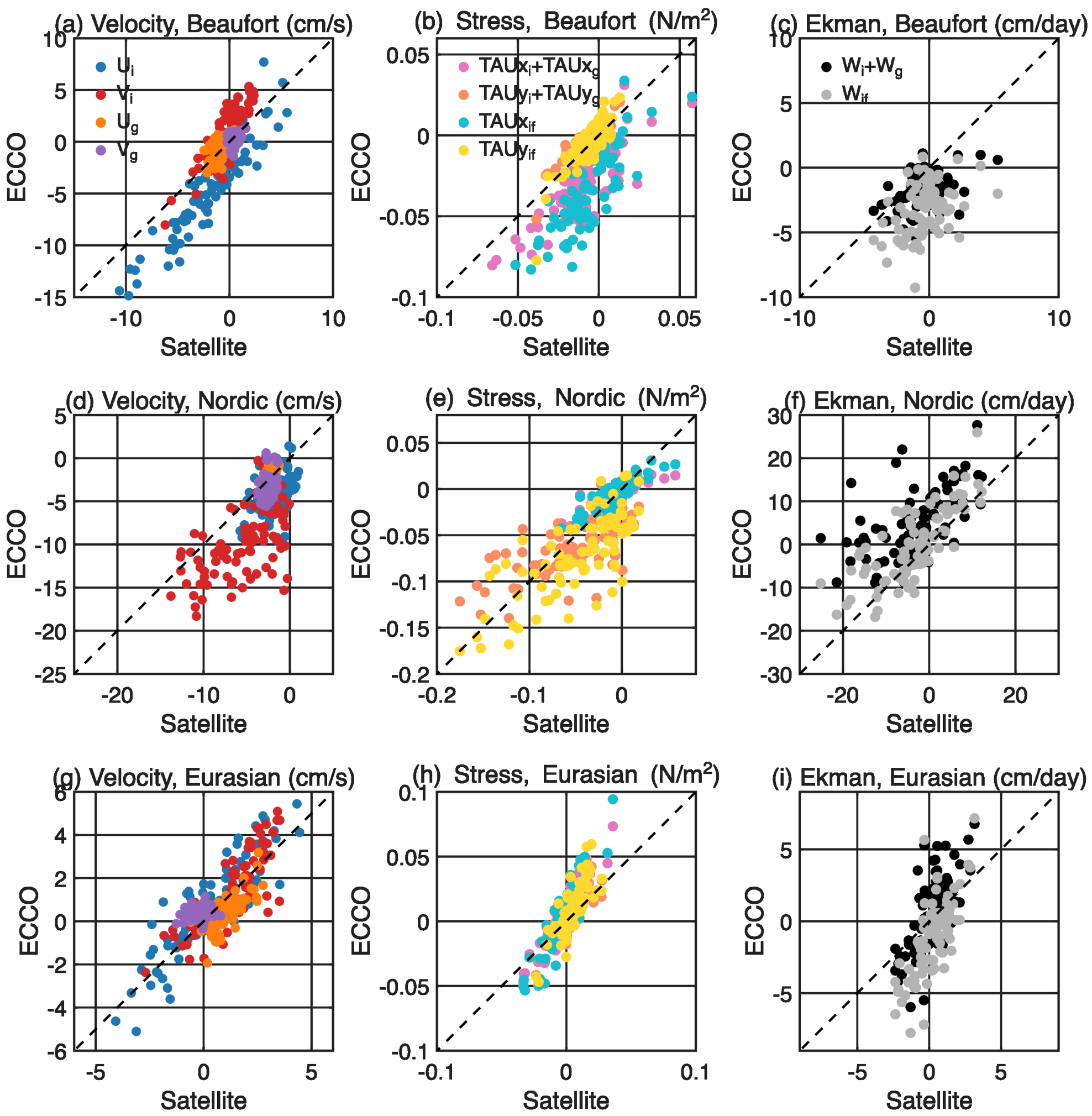

Figure S1. Monthly mean velocity, surface stress, and Ekman pumping rate in the Beaufort Gyre, Nordic Seas and Eurasian regions. (a) Zonal (U) and meridional (V) components of sea-ice motion and geostrophic velocity from satellite data and ECCO in the Beaufort region. Positive value means east/north flow; (b) U/V components of surface stress from satellite data and two ECCO estimates in the Beaufort region; (c) Ekman pumping rate estimated with satellite data and two ECCO estimations in the Beaufort region. Positive value means upwelling; (d-f) Same as (a-c) but for Nordic region; (g-i) Same as (a-c) but for Eurasian region.

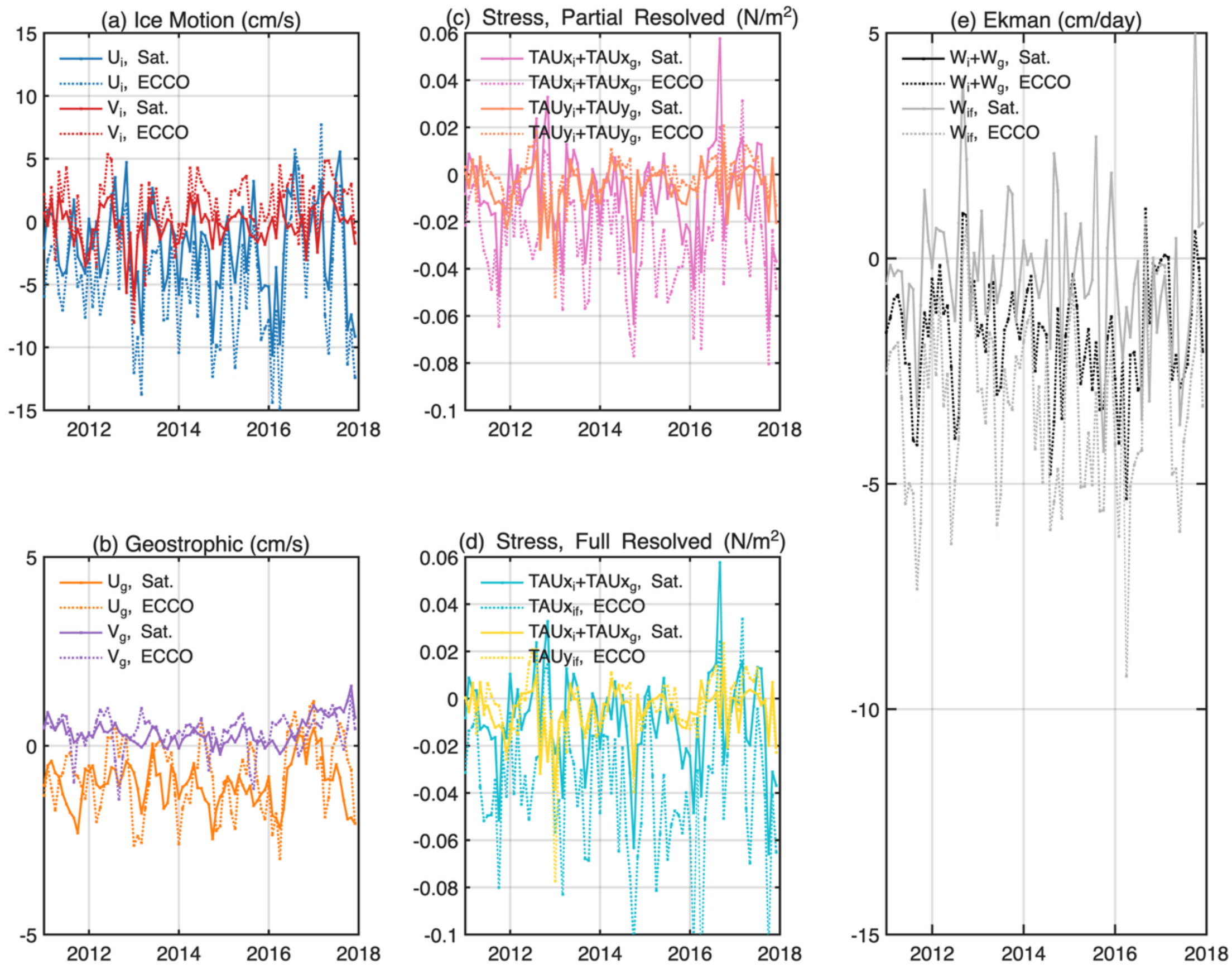

Figure S2. Timeseries of mean velocity, surface stress, and Ekman pumping rate in the Beaufort Gyre region. (a) Zonal (U) and meridional (V) components of sea-ice motion from satellite data and ECCO. Positive value means east/north flow; (b) U/V components of geostrophic velocity from satellite data and ECCO. Positive value means east/north flow; (c) U/V components of surface stress from satellite data and ECCO estimate (partial resolved); (d) same as (c), but for fully resolved estimate; (e) Ekman pumping rate estimated with satellite data and two ECCO estimations. Positive value means upwelling. For satellite estimates, $w_{if} = w_i + w_g$.

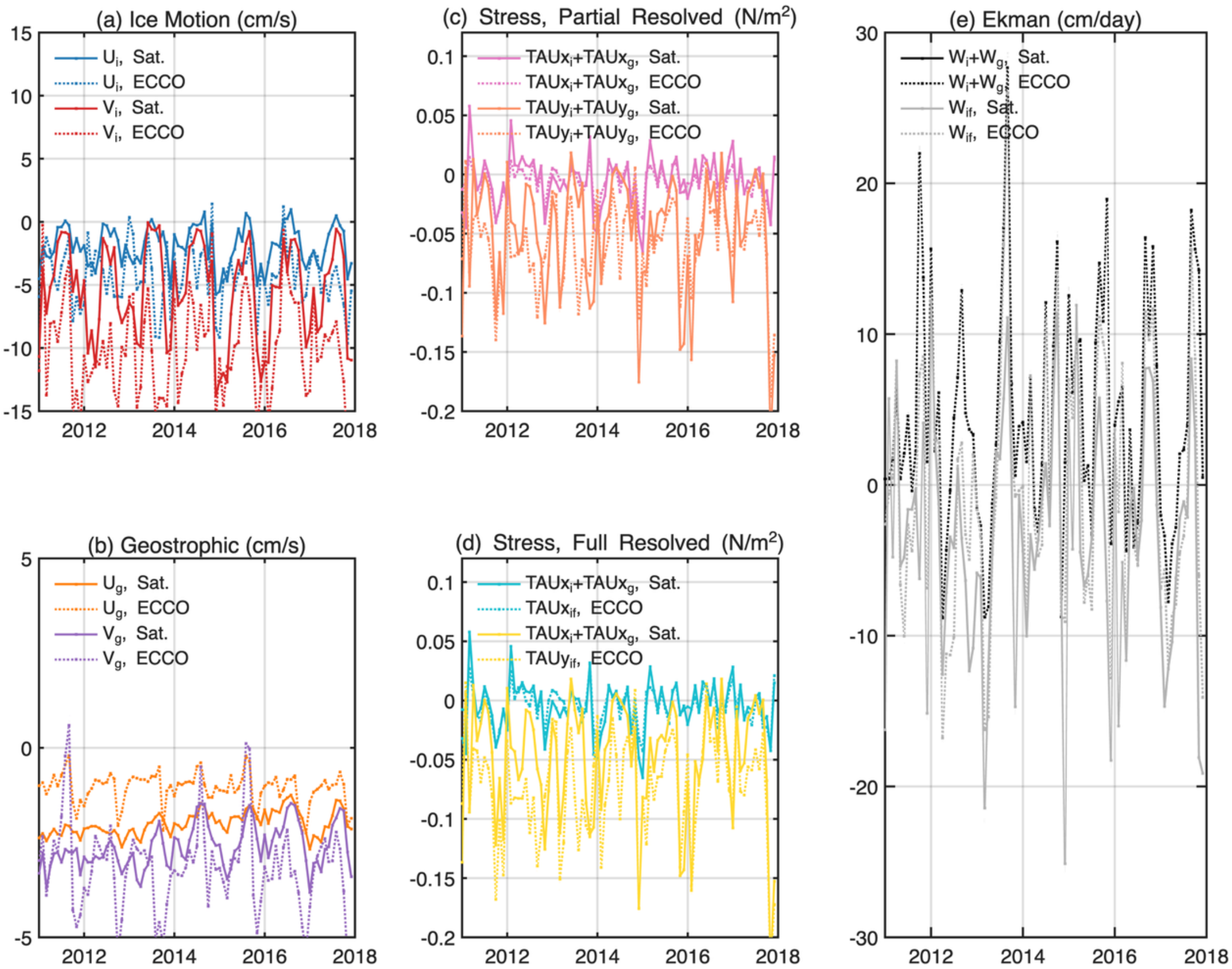

Figure S3. Same as Figure S2, but for the Nordic Seas region.

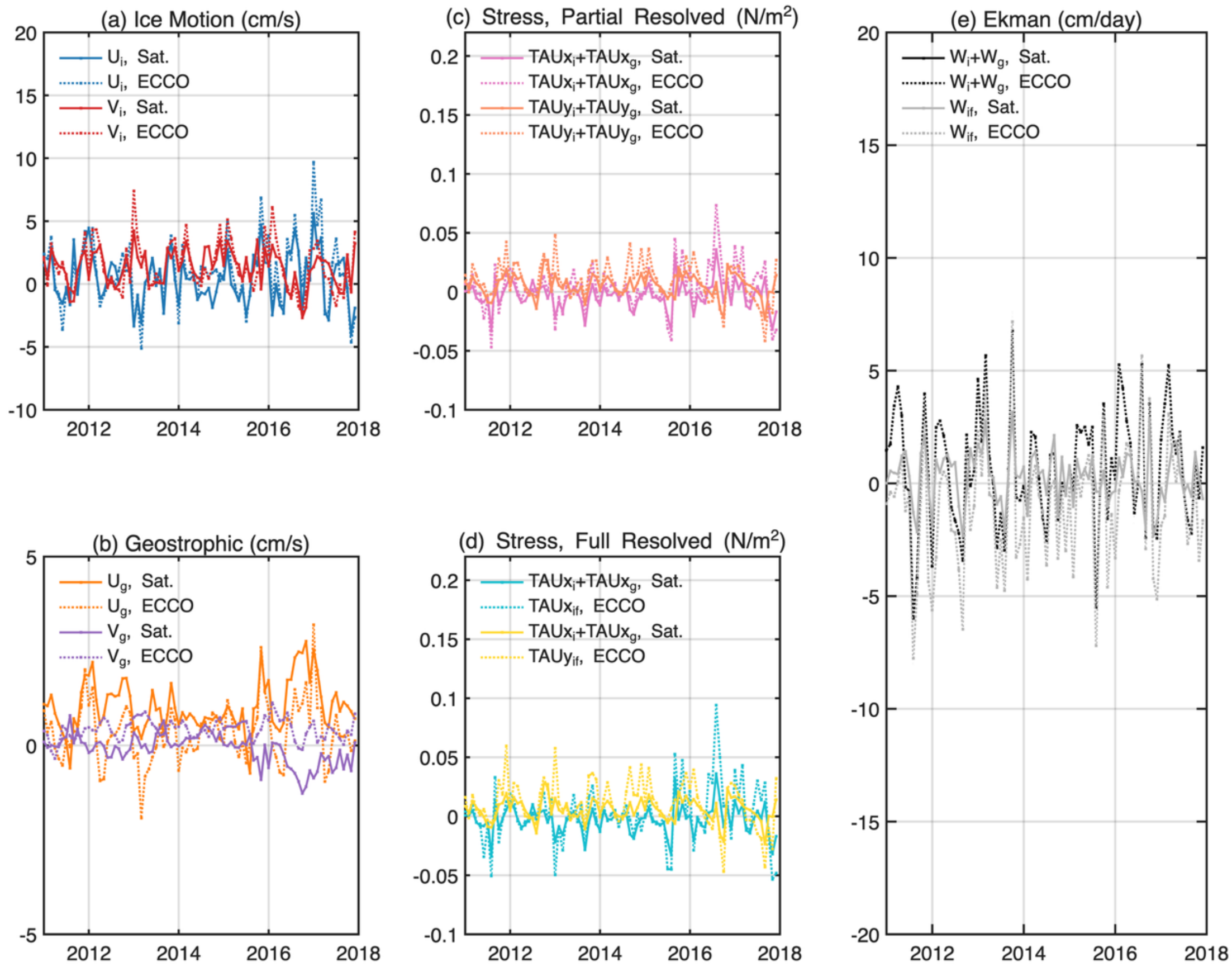

Figure S4. Same as Figure S2, but for the Eurasian region.

Table S1 Statistics of ECCO compared to satellite data. Correlations with p<0.01 are in bold. Standard Deviation Ratio (STD Ratio) is calculated as $X_{ECCO}/X_{Sat.}$. Units for Root-mean-square-error (RMSE; X_{ECCO} as estimated values, $X_{Sat.}$ as actual values) and mean bias (X_{ECCO}-X_{Sat}) are the same as corresponding variables.

Variable	Correlation	RMSE	STD Ratio	Mean Bias
Beaufort Gyre Region				
U_i	**0.92**	3.25	1.33	-2.59
U_g	**0.44**	0.90	1.55	0.23
$\tau_{x,i+g}$	**0.82**	0.02	1.11	-0.02
$\tau_{x,if}$	**0.85**	0.03	1.47	-0.03
w_{i+g}	**0.45**	2.02	0.83	-1.34
V_i	**0.87**	1.88	1.59	1.25
V_g	**0.24**	0.50	1.34	-0.01
$\tau_{y,i+g}$	**0.87**	0.01	1.05	0.00
$\tau_{y,if}$	**0.84**	0.01	1.45	0.00
w_{if}	**0.39**	4.48	1.26	-2.86
Nordic Seas Region				
U_i	**0.39**	3.24	1.43	-2.34
U_g	0.22	1.04	1.35	0.92
$\tau_{x,i+g}$	**0.86**	0.01	0.64	0.00
$\tau_{x,if}$	**0.88**	0.01	0.73	0.00
w_{i+g}	**0.58**	10.62	0.95	7.91
V_i	**0.56**	5.84	1.03	-4.61
V_g	0.26	1.27	2.02	-0.54
$\tau_{y,i+g}$	**0.75**	0.03	0.71	0.00
$\tau_{y,if}$	**0.78**	0.03	0.93	0.00
w_{if}	**0.76**	6.47	1.06	3.13
Eurasian Region				
U_i	**0.89**	1.23	1.26	0.46
U_g	**0.73**	0.88	1.17	-0.69
$\tau_{x,i+g}$	**0.93**	0.01	1.63	0.01
$\tau_{x,if}$	**0.91**	0.01	2.00	0.00
w_{i+g}	**0.67**	1.98	2.15	0.40
V_i	**0.82**	1.09	1.35	0.16
V_g	0.18	0.67	0.67	0.52
$\tau_{y,i+g}$	**0.81**	0.01	2.06	0.00
$\tau_{y,if}$	**0.81**	0.01	2.00	0.00
w_{if}	**0.73**	2.37	2.22	-0.13

Table S2 Statistics of Mean Ekman pumping rate associated with each forcing terms between the dynamic and stable months.

Forcing		Mean	Median	Min	Max	Interquartile
Beaufort Gyre Region						
Wind	Stable	-0.63	-0.05	-2.95	-0.03	0.75
	Dynamic	-0.92	-0.58	-2.37	0.05	2.03
Sea Ice	Stable	-356	-3.16	-5.35	-2.78	1.24
	Dynamic	-3.95	-3.89	-4.67	-3.27	1.11
Geostrophic	Stable	2.65	2.78	1.70	3.55	0.79
	Dynamic	2.74	2.64	2.24	3.62	0.65
Residual	Stable	-1.49	-1.40	-2.10	-0.97	0.68
	Dynamic	-2.58	-2.57	-3.75	-0.88	0.92
Nordic Seas Region						
Wind	Stable	5.56	5.71	4.33	7.00	1.97
	Dynamic	5.80	4.98	2.69	9.33	6.09
Sea Ice	Stable	5.21	6.25	1.05	6.55	1.90
	Dynamic	2.87	2.17	1.27	6.11	2.56
Geostrophic	Stable	-2.97	-2.84	-5.54	0.61	2.86
	Dynamic	-1.09	-0.34	-5.26	0.64	1.64
Residual	Stable	-1.95	-1.99	-2.92	-0.81	0.58
	Dynamic	-2.99	-3.03	-4.31	-2.10	0.44
Eurasian Region						
Wind	Stable	-0.27	-0.23	-0.57	-0.11	0.26
	Dynamic	0.15	-0.01	-0.24	1.45	0.07
Sea Ice	Stable	0.86	1.00	-0.08	1.33	0.67
	Dynamic	0.43	0.39	-0.29	1.10	0.75
Geostrophic	Stable	0.19	0.13	-0.64	0.83	1.06
	Dynamic	0.10	0.21	-1.00	1.32	1.63
Residual	Stable	-1.43	-1.57	-2.01	-0.47	0.75
	Dynamic	-0.75	-0.48	-1.72	-0.06	0.76

Mixed Layer Depth Variability

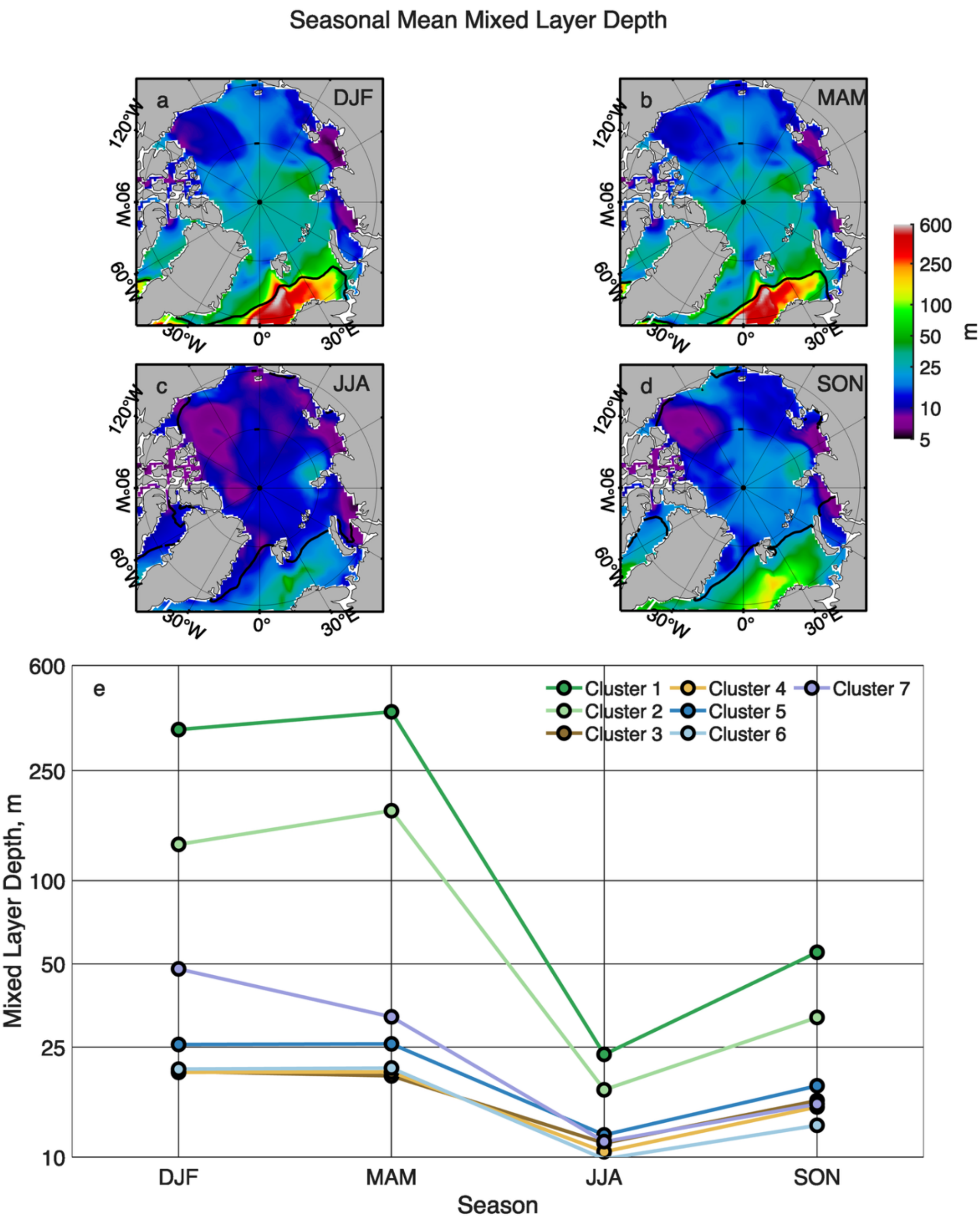

Figure S5. Mean mixed layer depth (MLD) in the Arctic basin. (a–d) Spatial distribution of mean MLD for in each season. (e) Seasonal mean MLD averaged over each cluster across the Arctic Ocean.

Robustness of GMM Clustering

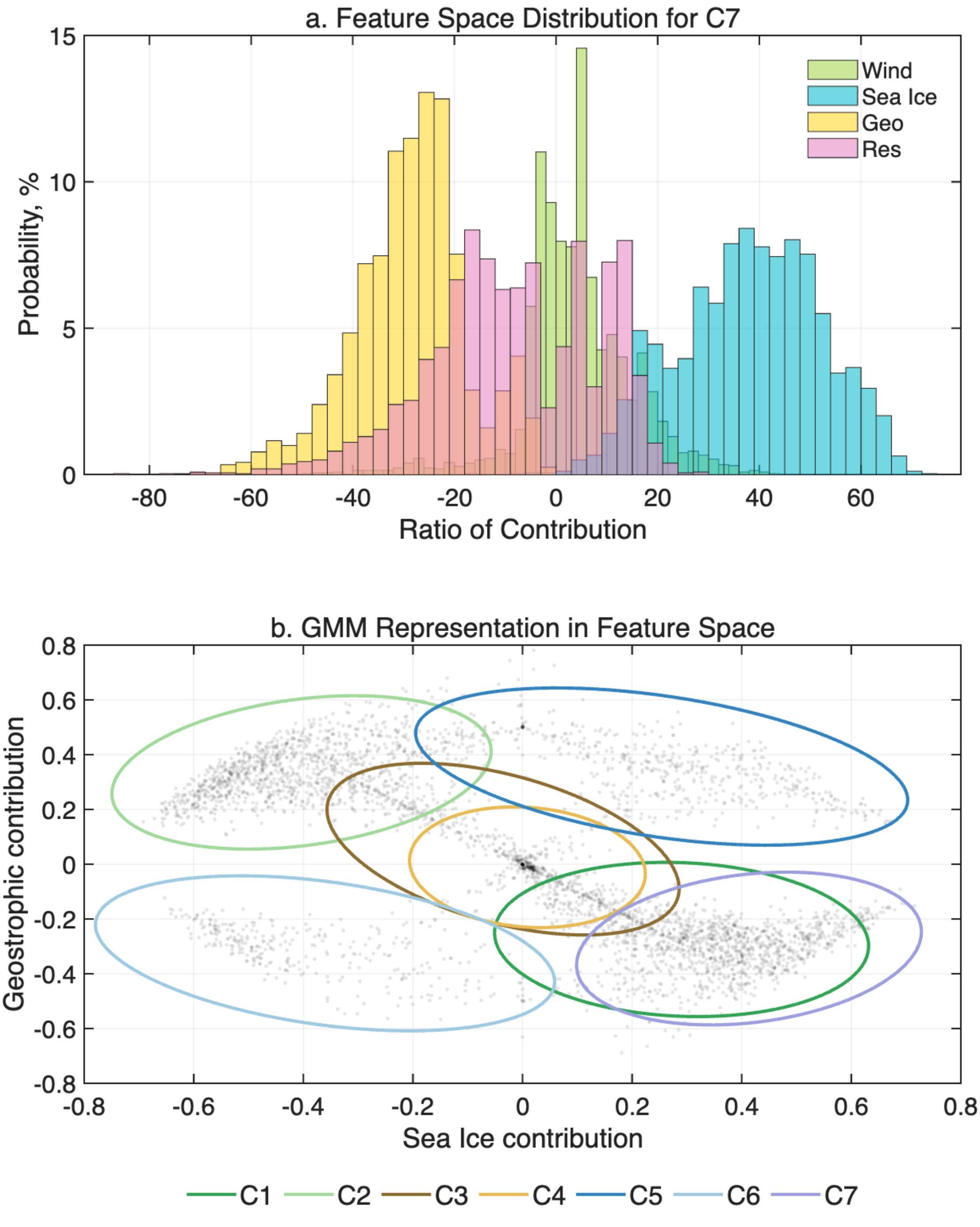

Figure S6. Distribution GMM clusters in feature Space. (a) Probability distribution of each feature for Cluster 7 (C7). (b) GMM representation in 2-dimension projected feature space. Dimensions of wind and residual contributions are not shown.

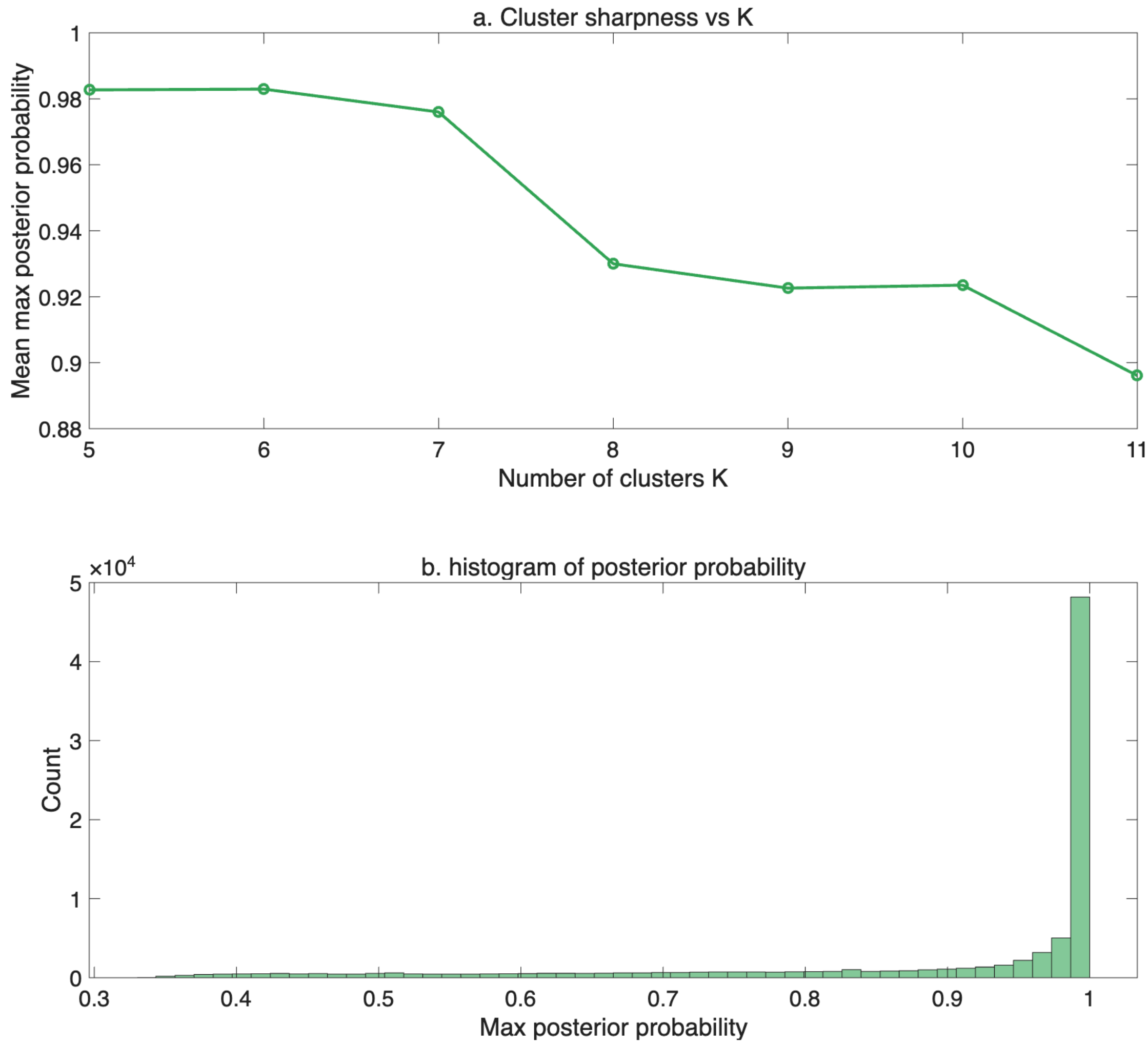

Figure S7. Cluster sharpness and assignment confidence as a function of model complexity. (a) Mean maximum posterior probability (cluster sharpness) as a function of the number of clusters K. (b) Distribution of maximum posterior probabilities for the selected model (K=7).

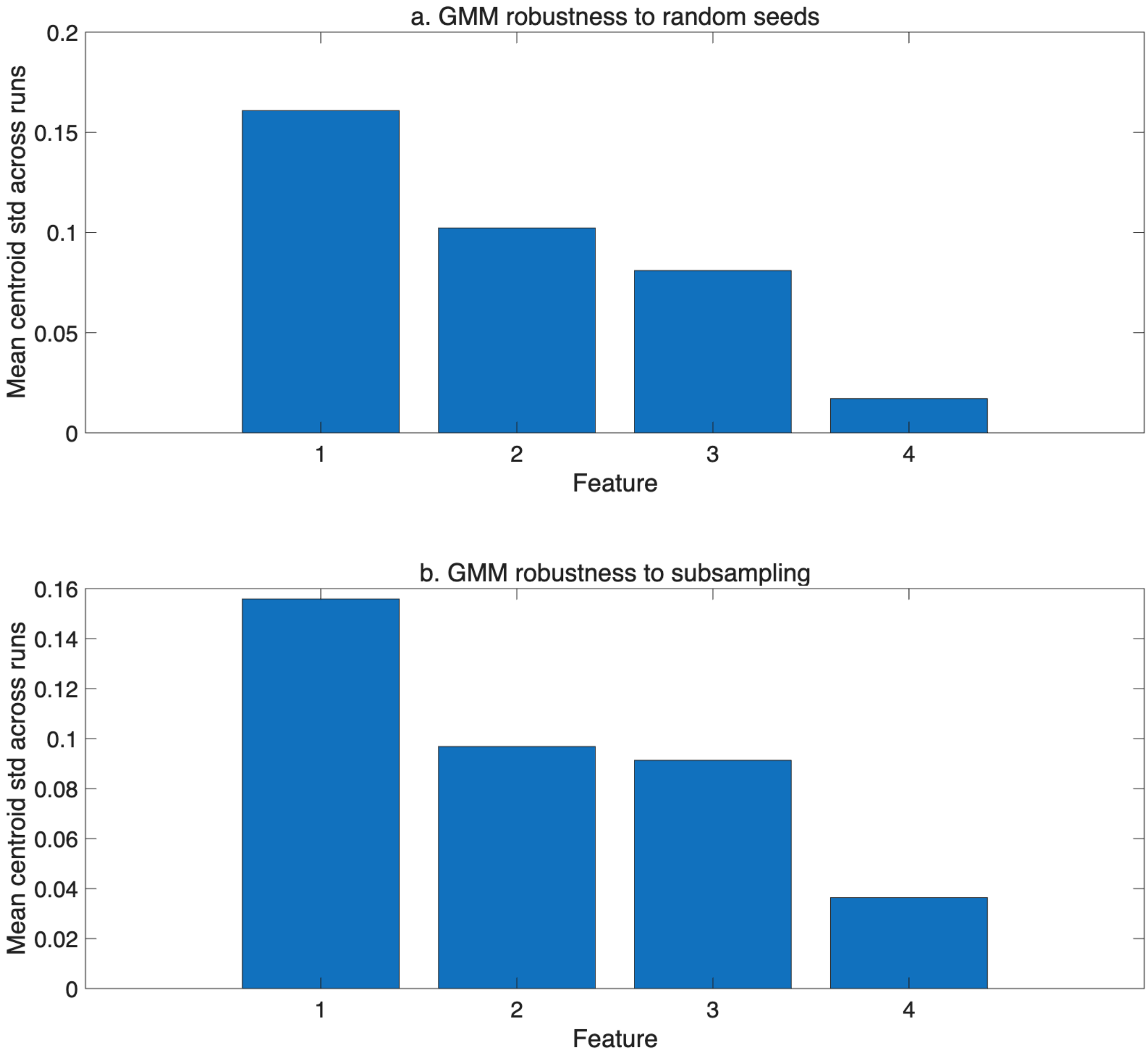

Figure S8. Robustness of GMM clustering to initialization and data subsampling. (a) Sensitivity of learned cluster structure to random initialization. Mean standard deviation of GMM cluster centroids (μ) across 10 independent model fits is shown for each feature. Lower values indicate more stable convergence of the clustering solution. (b) Sensitivity to input data subsampling. Mean standard deviation of cluster centroids is computed across 10 realizations in which a random fraction of the training dataset is withheld prior to model fitting, providing a measure of robustness to reduced data availability.
