# Supplementary material for: Regime transitions in Arctic surface momentum balance reveal persistent and predictable dynamical states
Source: Nat Commun. 2026 Jul 8;17:9132. doi: 10.1038/s41467-026-75328-7 (PMC13518975; doi:10.1038/s41467-026-75328-7)
Supplement: Supplementary file 2 [file 41467_2026_75328_MOESM2_ESM.pdf]

nature portfolio

Peer Review File

Regime Transitions in Arctic Surface Momentum Balance Reveal Persistent and Predictable Dynamical States

Corresponding Author: Dr Chao Liu

This file contains all reviewer reports in order by version, followed by all author rebuttals in order by version.

Version 0:

Reviewer comments:

Reviewer #1

(Remarks to the Author)
Review of “Regime Transitions in Arctic Surface Momentum Balance Reveal Persistent and Predictable Dynamical States” by Liu 2026

General Comments
This study investigates the changing air/ice-ocean momentum flux in the Arctic Ocean using output from a dynamically consistent ice-ocean coupled model (ECCOv4r4). The author employs a probabilistic machine-learning method to classify the momentum flux into seven distinct regimes, examining their seasonal and interannual variabilities. This is a timely and important topic, given the dramatic decline in Arctic sea ice in recent years. Rapid changes in air/ice-ocean interactions are expected to alter momentum fluxes, with potential effects on vertical heat, salt, and material exchanges. While the analysis offers some new insights, I have several major concerns regarding the validation of the model and the physical interpretation of the results.

1. Discrepancies between Model and Observations
My primary concern is that the outputs and derivatives (surface stress, Ekman pumping, etc.) from ECCOv4r4 show substantial discrepancies when compared to observation-based estimates. As shown in supplementary Figure S1, the differences are far from negligible. Furthermore, the significance levels for the correlation coefficients between the model and observations are notably absent. A more rigorous comparison is necessary. The author should evaluate the timeseries of these metrics not just in terms of mean magnitudes, but also regarding their temporal coherence and variability to justify the model's reliability for this specific application.

2. Physical Interpretation and Vertical Coupling
Regarding the broader implications of the seven dynamical regimes, the discussion links to vertical redistribution of heat and freshwater are somewhat superficial. It is difficult to determine from the current text how these defined surface regimes specifically alter subsurface properties. One cannot simply use these surface dynamic regimes to predict how weakened ice cover will alter Arctic-wide mixing and stratification. The metrics analyzed (surface stress) are consequences of surface dynamics (ice motion, concentration, geostrophic currents, wind), but they do not account for the stratification barrier. Therefore, the manuscript discusses the “drivers” of momentum flux without establishing a direct, quantified link to the “consequences” for vertical exchange.

Specific Comments
Lines 20–21: The conclusion stated here is not immediately evident from the results presented in the main text (specifically related to Lines 171–172 and Figure 3g). Please clarify the basis for this statement.
Line 73: The regions defined by the boxes in Figure 1e are significantly larger than the standard geographical definitions of the “Beaufort Sea” and “Fram Strait”. It would be more accurate to label these as the “Beaufort Gyre region” and the “Nordic Seas” (or “North Atlantic”).
Lines 111–112: The phrase “the same sense” is ambiguous. Does this refer to the same mean ratio of contribution as shown in Figure 2g? Please clarify.
Line 133: Typo: the “upwelling” should be “downwelling” in this context.
Lines 151–153: The rationale for selecting only the “Beaufort Sea” and “Fram Strait” requires stronger justification. The Eurasian Basin is also experiencing rapid changes in ocean circulation and sea ice, excluding it limits the scope of the

assessment regarding Arctic-wide changes.
Lines 171–172: I am unable to derive the stated information from Figure 3g. Please verify the figure reference or expand the explanation to make the connection clear to the reader.
Lines 182–183: To improve readability, please explicitly list the specific categories here using their defined names/numbers. Similar broad descriptions occur elsewhere in the text without direct links to the specific figures or the seven categories, making it difficult to follow the argument.
Lines 198–199: Is the residual term truly the only component showing a statistically significant decrease? Visual inspection of Figure 3h suggests that the changes in sea ice and geostrophic components are also significant. Please provide the corresponding mean values and variance statistics to support this claim.
Lines 213–214: The statement that winter is characterized by "minimum surface buoyancy forcing" is questionable. Fall and winter are typically associated with the strongest brine rejection due to sea ice formation, which is a major buoyancy forcing mechanism. Please revise or clarify this physical reasoning.
Lines 241–242: Please provide quantitative definitions for the terms "dynamically active years" and "stable periods" as used in this analysis.
Lines 257–258: Which specific figures support the conclusion that "Residual-dominated regimes are systematically associated with ice-ocean balanced states"?
Lines 265–267: Is this specific case observed within your defined "Fram Strait" region?
Lines 269–271: The discussion here lacks depth. An increase in wind power input does not automatically lead to vertical mixing if the stratification barrier remains strong enough to suppress it. The manuscript should acknowledge the competition between increased surface stress and potential changes in stratification (e.g., Guthrie et al., 2013; Lincoln et al., 2016).
Lines 277–279: Are these statements referring to the patterns shown in Figures 4a and 4c? Please clarify.
Figure 1a: Typo in the title: It should be "DJF" (December-January-February) instead of "DJM".
Figure 4e: Cluster C2 appears to be missing from the timeseries in Figure 4e.

References
Guthrie, J. D., Morison, J. H., & Fer, I. (2013). Revisiting internal waves and mixing in the Arctic Ocean. Journal of Geophysical Research, 118, 3966–3977. https://doi.org/10.1002/jgrc.20294
Lincoln, B. J., Rippeth, T. P., Lenn, Y. D., Timmermans, M. L., Williams, W. J., & Bacon, S. (2016). Wind-driven mixing at intermediate depths in an ice-free Arctic Ocean. Geophysical Research Letters, 43, 9749–9756. https://doi.org/10.1002/2016GL070454

Reviewer #2

(Remarks to the Author)
Dear editor and authors,

This novel analysis uses unsupervised machine learning classification to identify distinct physical regimes of the Arctic surface momentum balance. Many of the conclusions are important and point to particular combinations of underlying mechanisms.

The authors highlight the potential role of "internal stress divergence" of sea-ice as a contributor to the surface momentum balance. This may very well be the case in the real world. However, ECCOv4 is not the appropriate tool to identify the role of "internal stress divergence" of sea-ice, as ECCOv4 does not incorporate that in the momentum transfer between the ice and the ocean. I think the authors should therefore be more cautious in describing their important "residual" momentum component. In fact, I think an updated version of the manuscript should carefully consider the potential contributions to this residual component in greater detail. Because of the importance of this issue, I recommend Major Revisions.

Major comments:

Lines 259-260: You list "unresolved processes such as internal stress divergence, small-scale deformation, and variable ice-ocean drag" - Actually, ECCOv4 does not use internal stress divergence and deformation of sea-ice when calculating the ice-ocean drag. ECCOv4 uses the Campin et al. (2008) formulation of ice-ocean drag rather than the Hibler and Bryan (1987) internal stress model.

So out of your list above, only the high-frequency ice-ocean covariability actually contributes to the residual term in ECCO. You should correct your statement.

Lines 290-292: "All non-residual momentum contributors -- surface wind stress... -- are directly observed or derived from satellite measurements, grounding the identified regimes in quantities that are routinely monitored..." - Please comment whether surface wind stress is accurately estimated in zones of large sea-ice concentration.

Minor comments:

Figure 1 caption, lines 68-73: specify the sign convention, e.g., "negative downward"

Lines 111-111: "with both terms acting in the same sense within each regime" - express more clearly the conclusion that the

two terms are of the same sign.

Figure 3 caption, lines 142-149: specify the sign convention, e.g., "positive upward"

Figure 3, panels a and b, legend: it is a bit hard to distinguish the colors indicating C1 and C5, C2 and C6. Could you use markers of different shapes to distinguish among them?

Figure S1 caption, Line 43 in the Supplementary Information: "from satellite data" - specify what satellite data. What is the data source?

Additional references:

J.-M. Campin, J. Marshall, and D. Ferreira. Sea ice–ocean coupling using a rescaled vertical coordinate z*. Ocean Modelling, 24(1–2):1–14, 2008. doi:10.1016/j.ocemod.2008.05.005.

W. D. Hibler, III and K. Bryan. A diagnostic ice-ocean model. J. Phys. Oceanogr., 17(7):987–1015, 1987. DOI: https://doi.org/10.1175/1520-0485(1987)017<0987:ADIM>2.0.CO;2

Reviewer #3

(Remarks to the Author)
Review of “Regime Transitions in Arctic Surface Momentum Balance Reveal Persistent and Predictable Dynamical States” by Chao Liu
This manuscript explores the relative contribution of decomposed Ekman pumping components on Arctic surface momentum balance using the ECCO reanalysis product and Gaussian Mixture Modelling to identify distinct Ekman pumping regimes around the Arctic. The study adds to a long line of work studying ocean dynamics using unsupervised clustering methods (e.g., Sun et al. 2020, Jones et al., 2019). However, while much past work has focussed on clustering the ocean state using thermohaline properties, these authors cluster specifically on Ekman pumping components, enabling a direct connection between the GMM components and Ekman pumping contributors.
The work is well written, with an easy-to-follow method and clear results. However, given the volume of past GMM work, I was expecting some more details to the clustering method used. In addition, given the sensitivity of GMM to the input data used, I was unsure about the applicability of the results to a broader set of observational or modelling works. I recommend publication of this work after substantial rework of the manuscript based on the following points:
1) More clarity on the GMM method
a) The authors do not make a convincing case for why GMM is the most appropriate clustering method in this work. While I agree that deterministic methods like K-means are not appropriate given the spatiotemporal mixing and variability between clusters, I am not sure fitting the w components to Gaussians is more appropriate. Gaussians can be a better choice for distributions that have approximately Gaussian spreads, but it is unclear if the underlying training data here is approximately Gaussian. I suggest the authors provide a step-by-step illustration of the underlying distribution, and the fitted Gaussians, to reassure the reader that GMM is an appropriate method for the underlying training data.
b) Similarly, I was unsure why $K=7$ clusters ended up being used as the optimal number of clusters. I understand that the AIC and BIC begin to change minimally for more than 7 clusters, which can provide a statistical basis for this choice. However, given your end-use is to understand ocean dynamics and Ekman pumping regimes, I feel that a somewhat dynamical explanation may be more convincing. For example, if you could show that, when some seasons are omitted from the GMM training, the ability of GMM to represent the key clusters is unaffected for $K>7$, that would be interesting and convincing. Or, if you could show that the PDF in space for each cluster (i.e., the probability obtained from GMM) begins to sharpen at $K>7$, then you could say that you have minimised the presence of “mixed” boundaries between clusters.
c) GMM is typically sensitive to an initial “random seed” – i.e., where the initial Gaussians are generated in phase space before iteratively moving to the final distribution/cluster location. This sensitivity should be explored in the work. If the random seed is modified, what do the distributions look like? Do they change a lot? What about the probability associated with the clusters?
d) Figures 2 and 3: The assignment of clusters via GMM is determined purely by the fact that $p>0.5$ for a given cluster. Hence, the assessments in Fig 2 and 3 are somewhat misleading, as vast proportions of the clusters could have relatively mixed probabilities, e.g. $0.5<p<0.6$. To show this, the diagnostic in Figs. 2 and 3 should also be shown for clusters defined by $p>0.9$, for example. This will show how much influence “mixed” regions have on these diagnostics (like ocean area ratio).

2) Applicability of method to alternative data sources
I was unsure of how robust this GMM method would be to different data sources. For example, if the GMM is highly sensitive to the nature of the input data, then these results may only hold true for ECCO and break down for other models or observational datasets. This would substantially weaken the conclusions, in my view. I encourage the authors to consider some alternative approaches.
Some options include:
a) Remove some training data randomly (spatially or temporally) and evaluate the robustness of the GMM results to this reduced training dataset.
b) Subsample training data to align with available observations and see how robust the GMM clusters are.
c) Remove future years or seasons and see if the trained GMM can fit well over these years or seasons (to assess the impact of seasonal biases in observations on results).
d) Assess other reanalysis or modelling datasets to determine the robustness of the results of different ocean-sea ice

physics and forcing fields.
Minor Comments:

- How does the author compute each Ekman pumping contribution for each one of the clusters (fig 2 g).
- The author talks about “a critical link between atmospheric forcing and the vertical redistribution of heat and freshwater, offering a roadmap for predicting how weakened ice cover will alter arctic-wide mixing and stratification” in the abstract. However, I was unable to find any mention of vertical distributions or stratification again in the discussion. The only mention was a brief discussion around the mixed layer.

Minor Changes
- line 107 areal -> area
- fig 1 (f) -> (d).

Reviewer #4

(Remarks to the Author)
I co-reviewed this manuscript with one of the reviewers who provided the listed reports. This is part of the Nature Communications initiative to facilitate training in peer review and to provide appropriate recognition for Early Career Researchers who co-review manuscripts.

Version 1:

Reviewer comments:

Reviewer #1

(Remarks to the Author)
I appreciate the authors for their clarification and multiple modifications. The limitation of the study is now explicit presented. However, I was not clear enough, not just the temporal coherence and variability of ECCOv4r4 is different with the observational based method, but also their spatial patterns (in terms of your regime structure). This bias should also be mentioned.
In the abstract, you concluded “Spatially, we observe in the Beaufort and other marginal seas an increasing frequency of seasonal transitions from stable geostrophic-controlled states toward wind-driven downwelling.” I think this is related to lines 271-272 and lines 280-290 (Figures 4a-c), what I see is an increasing frequency of cluster 5 (C5), and this cluster reflects “ice drift induces Ekman upwelling, which is partially compensated by geostrophic downwelling” (in lines 141-142). This does not make sense to your claim that “increase seasonal transitions from stable geostrophic-controlled states toward wind-driven downwelling”. The wind-driven downwelling regime corresponds to cluster 2 (C2), which exhibits persistently zero values throughout the study periods (Figure 4c)!

Reviewer #2

(Remarks to the Author)
Dear editor and authors,

This version of the manuscript is substantially improved, and the authors have addressed all my previous comments. I therefore recommend accepting this manuscript for publication in its present form.

Reviewer #3

(Remarks to the Author)
Thank you to the authors for so comprehensively addressing the technical concerns raised in our review. We are happy with the state of the manuscript, and recommend publication based on its technical merits.

Reviewer #4

(Remarks to the Author)
I co-reviewed this manuscript with one of the reviewers who provided the listed reports. This is part of the Nature Communications initiative to facilitate training in peer review and to provide appropriate recognition for Early Career Researchers who co-review manuscripts.

Version 2:

Reviewer comments:

Reviewer #1

(Remarks to the Author)
This is my third-round review of Liu and Liang 2026. My evaluation is that their study brings new insight into the changing dynamic regime of Ekman pumping in the Arctic Ocean. But I still don’t follow some of the arguments that were presented in

the paper. For their response “For the Beaufort Sea (Figures 4a), the primary increase is associated with Cluster 5 & 6 (C5 & C6), as the 5th & 6th columns are mostly marked with higher probability.”, I only see an increase in Cluster 5 in Figure 4c. The Cluster 4 shows an increase until 2006-2007, and then it gradually decreased (Figure 4c). The mentioned Figure 4a only shows the probability of transition from one cluster to another cluster, but it does not imply the trend (increase).

In another response, “In contrast, the Nordic/Eurasian sector (Figures 4b & 4c) exhibits increasing occurrence and persistence of Cluster 1 (C1) and Cluster 2 (C2)”, the Nordic/Eurasian sector does not refer to your Figures 4b & 4c! And I don’t see Cluster 1 and Cluster 2 exhibit increasing occurrence, what I see from Figures 4f & 4i are persistence of Cluster 1 and Cluster 2 rather than increasing. How do you conclude that the Nordic/Eurasian sector exhibits an increasing wind-driven downwelling regime?

The revised conclusion in the abstract, “Spatially, the Beaufort Sea shifts from persistent geostrophic-balanced states toward more dynamically active ice–geostrophic regimes, whereas the Nordic/Eurasian marginal seas exhibit increasing prevalence of wind-driven downwelling states.” was not even mentioned in the main text.

My last suggestion is to be more careful in interpreting your results, and please do not rush to submit the revision.

Version 3:

Reviewer comments:

Reviewer #1

(Remarks to the Author)
The authors had addressed my comments. I recommend the manuscript for acceptance.

Open Access This Peer Review File is licensed under a Creative Commons Attribution 4.0 International License, which permits use, sharing, adaptation, distribution and reproduction in any medium or format, as long as you give appropriate credit to the original author(s) and the source, provide a link to the Creative Commons license, and indicate if changes were made.
In cases where reviewers are anonymous, credit should be given to 'Anonymous Referee' and the source.
The images or other third party material in this Peer Review File are included in the article’s Creative Commons license, unless indicated otherwise in a credit line to the material. If material is not included in the article’s Creative Commons license and your intended use is not permitted by statutory regulation or exceeds the permitted use, you will need to obtain permission directly from the copyright holder.
To view a copy of this license, visit https://creativecommons.org/licenses/by/4.0/

Reviewer #1 (Remarks to the Author):

Review of “Regime Transitions in Arctic Surface Momentum Balance Reveal Persistent and Predictable Dynamical States” by Liu 2026

General Comments

This study investigates the changing air/ice-ocean momentum flux in the Arctic Ocean using output from a dynamically consistent ice-ocean coupled model (ECCOv4r4). The author employs a probabilistic machine-learning method to classify the momentum flux into seven distinct regimes, examining their seasonal and interannual variabilities. This is a timely and important topic, given the dramatic decline in Arctic sea ice in recent years. Rapid changes in air/ice-ocean interactions are expected to alter momentum fluxes, with potential effects on vertical heat, salt, and material exchanges. While the analysis offers some new insights, I have several major concerns regarding the validation of the model and the physical interpretation of the results.

Thank you for this important assessment. We agree that both model validation and physical interpretation are critical for the robustness of this study. In the revised manuscript, we have (1) strengthened the validation of ECCO-derived quantities through additional comparisons with observation-based estimates, including time series and correlation analyses, and (2) revised the interpretation of the results to avoid overstatement, particularly regarding the role of the residual term and its physical implications. These changes are described in detail below.

1. Discrepancies between Model and Observations

My primary concern is that the outputs and derivatives (surface stress, Ekman pumping, etc.) from ECCOv4r4 show substantial discrepancies when compared to observation-based estimates. As shown in supplementary Figure S1, the differences are far from negligible. Furthermore, the significance levels for the correlation coefficients between the model and observations are notably absent. A more rigorous comparison is necessary. The author should evaluate the timeseries of these metrics not just in terms of mean magnitudes, but also regarding their temporal coherence and variability to justify the model's reliability for this specific application.

Thanks for this important point. We agree that a more rigorous validation is necessary to justify the use of ECCOv4r4 for this analysis.

In the revised manuscript, we have strengthened the comparison with observation-based estimates in three ways. First, we now include time series comparisons of key quantities (Ekman pumping and surface stress) at regional scales (**Fig. S2-S4**), allowing direct assessment of temporal coherence and variability. Second, we report correlation coefficients together with their statistical significance (p-values),

demonstrating that the model captures a significant fraction of the observed variability (**Table S1**).

While differences in magnitude remain, we now explicitly acknowledge these biases in the text (**Lines 399-403**) and clarify that our analysis focuses on the relative contributions and regime structure, which depend more strongly on variability and co-variability than on absolute magnitudes. The revised discussion highlights these limitations and their implications for interpretation.

2. Physical Interpretation and Vertical Coupling
Regarding the broader implications of the seven dynamical regimes, the discussion links to vertical redistribution of heat and freshwater are somewhat superficial. It is difficult to determine from the current text how these defined surface regimes specifically alter subsurface properties. One cannot simply use these surface dynamic regimes to predict how weakened ice cover will alter Arctic-wide mixing and stratification. The metrics analyzed (surface stress) are consequences of surface dynamics (ice motion, concentration, geostrophic currents, wind), but they do not account for the stratification barrier. Therefore, the manuscript discusses the "drivers" of momentum flux without establishing a direct, quantified link to the "consequences" for vertical exchange.

We agree that the original manuscript overstated the connection between surface momentum regimes and vertical redistribution, and did not sufficiently account for the role of stratification in modulating vertical exchange.

In the revised manuscript, we have taken two steps to address this issue. First, we have moderated the interpretation and removed statements that imply a direct causal link between surface regimes and basin-scale mixing or stratification changes. **(Lines 366-378)**

Second, to provide a more physically grounded connection, we have introduced a brief discussion based on mixed layer depth (MLD) from ECCO. We show that different dynamical regimes are associated with systematically different MLD distributions (**Fig. S5)**, indicating that these surface momentum states correspond to distinct upper-ocean vertical structures.

While this does not establish a direct causal relationship, it provides a quantitative link between surface forcing regimes and upper-ocean vertical variability. We emphasize that the role of stratification in mediating vertical exchange remains an important avenue for future work.

Specific Comments
Lines 20–21: The conclusion stated here is not immediately evident from the results

presented in the main text (specifically related to Lines 171–172 and Figure 3g). Please clarify the basis for this statement.

We thank the reviewer for pointing out this lack of clarity. In the original manuscript, the connection between the residual term and periods of rapid sea-ice transition was not stated explicitly enough to support the conclusion in the Abstract.

Here, "intensification" refers to both (i) an increase in the absolute magnitude of the residual Ekman pumping and (ii) its enhanced relative contribution to the total momentum balance during periods of rapid sea-ice change.

We have revised the manuscript to clarify this linkage. In particular, in the Beaufort Gyre region (Fig. 3b), the residual magnitude peaks during June and October, coinciding with periods of rapid ice melt and freeze-up. In addition, its relative contribution increases during these transition periods as the geostrophic and ice-driven components weaken. Similar behavior is observed in the Nordic Seas and Eurasian Basin.

We have revised the relevant text (**Lines 21-23**) to explicitly describe this relationship and to guide the reader to the supporting figures.

Line 21-23: "Crucially, we identify a significant residual momentum component that is enhanced during seasonal sea-ice transitions, both in magnitude and in its relative contribution to the total momentum balance. "

Line 73: The regions defined by the boxes in Figure 1e are significantly larger than the standard geographical definitions of the "Beaufort Sea" and "Fram Strait". It would be more accurate to label these as the "Beaufort Gyre region" and the "Nordic Seas" (or "North Atlantic").

We have revised the terminology accordingly for accuracy. Thanks!

Lines 111–112: The phrase "the same sense" is ambiguous. Does this refer to the same mean ratio of contribution as shown in Figure 2g? Please clarify.

We have clarified this statement by explicitly referring to the composition of the contributions.

Lines 129-131: "Clusters C3 and C4 are characterized as sea-ice–geostrophic cooperative regimes, in which the ice and geostrophic contributions act in the same direction, with C3 and C4 differing primarily by a reversal in sign."

Line 133: Typo: the "upwelling" should be "downwelling" in this context.

It is corrected (**line 152**). Thanks!

Lines 151–153: The rationale for selecting only the “Beaufort Sea” and “Fram Strait” requires stronger justification. The Eurasian Basin is also experiencing rapid changes in ocean circulation and sea ice, excluding it limits the scope of the assessment regarding Arctic-wide changes.

We have clarified that these regions are selected as representative examples of contrasting dynamical regimes (persistent vs transitional), and we now explicitly discuss the Eurasian Basin (**revised Figures 3 & 4, Figures S1-S4, and Table S1, Lines 227-249**).

Lines 171–172: I am unable to derive the stated information from Figure 3g. Please verify the figure reference or expand the explanation to make the connection clear to the reader.

We have revised the figure reference and expanded the explanation to ensure the connection is clear (**Lines 196-199**).

Lines 182–183: To improve readability, please explicitly list the specific categories here using their defined names/numbers. Similar broad descriptions occur elsewhere in the text without direct links to the specific figures or the seven categories, making it difficult to follow the argument.

We have revised the text to explicitly reference cluster numbers and categories (e.g., **Lines 172, 201, 208**).

Lines 198–199: Is the residual term truly the only component showing a statistically significant decrease? Visual inspection of Figure 3h suggests that the changes in sea ice and geostrophic components are also significant. Please provide the corresponding mean values and variance statistics to support this claim.

Thank you for this important comment.

Across all three regions examined, we find that while the wind, sea-ice, and geostrophic contributions exhibit modest shifts in their mean values, these changes are generally not statistically significant at the 95% confidence level. In contrast, the residual component shows a robust and statistically significant change ($p < 0.05$) in all examined regions (including Eurasian). The statistics for Figure 3 are summarized in **Table S2**.

We have revised the manuscript (e.g., **Lines 244-249**) to (i) report the mean and variance changes for all components, (ii) include the Eurasian Basin in the regional

comparison, and (iii) clarify that the residual term is the only component exhibiting a statistically significant change, rather than the only component that varies.

Lines 213–214: The statement that winter is characterized by "minimum surface buoyancy forcing" is questionable. Fall and winter are typically associated with the strongest brine rejection due to sea ice formation, which is a major buoyancy forcing mechanism. Please revise or clarify this physical reasoning.

Thank you for this important clarification. We agree that winter is not a period of minimal buoyancy forcing, as brine rejection during sea-ice formation represents a significant source of buoyancy flux.

We have revised the manuscript to clarify that our focus on winter is not due to an absence of buoyancy forcing, but rather due to the reduced complexity of competing surface processes, particularly the absence of melt-related freshwater input and enhanced mechanical forcing from wind and sea-ice motion (**Lines 261-265**).

Lines 261-265: "Here we focus on wintertime transitions of the dynamical regimes, as this period is characterized by relatively stable surface conditions and reduced complexity from melt-related freshwater inputs. This allows wind- and ice-driven momentum transfer to be more clearly interpreted in the Ekman response, despite the presence of buoyancy forcing associated with brine rejection during sea-ice formation."

Lines 241–242: Please provide quantitative definitions for the terms "dynamically active years" and "stable periods" as used in this analysis.

The statement is revised for clarity (**Lines 295-296**).

Lines 295-296: "In the Nordic Seas, interannual variability in regime persistence ranges from approximately 50–60% at its lowest to nearly 80% at its highest."

Lines 257–258: Which specific figures support the conclusion that "Residual-dominated regimes are systematically associated with ice-ocean balanced states"?

Thank you for highlighting this point. We have revised the corresponding discussion to avoid over-interpretation (**Lines 302–309**).

Lines 265–267: Is this specific case observed within your defined "Fram Strait" region?

It is related to the Fram Strait region. This paragraph is largely revised and the statement is removed from the revision.

Lines 269–271: The discussion here lacks depth. An increase in wind power input does not automatically lead to vertical mixing if the stratification barrier remains strong enough to suppress it. The manuscript should acknowledge the competition between increased surface stress and potential changes in stratification (e.g., Guthrie et al., 2013; Lincoln et al., 2016).

We agree and have revised this section to acknowledge that increased surface stress does not necessarily translate to enhanced vertical mixing due to the presence of strong stratification. We now explicitly discuss this limitation and cite relevant studies (**Lines 366–378**).

Lines 277–279: Are these statements referring to the patterns shown in Figures 4a and 4c? Please clarify.

Thanks for pointing out. It is and we have clarified this statement (**Line 325**).

Figure 1a: Typo in the title: It should be “DJF” (December-January-February) instead of “DJM”.

It is corrected. Thanks!

Figure 4e: Cluster C2 appears to be missing from the timeseries in Figure 4e.

We have corrected the figure to include Cluster C2.

References

Guthrie, J. D., Morison, J. H., & Fer, I. (2013). Revisiting internal waves and mixing in the Arctic Ocean. Journal of Geophysical Research, 118, 3966–3977. https://doi.org/10.1002/jgrc.20294

Lincoln, B. J., Rippeth, T. P., Lenn, Y. D., Timmermans, M. L., Williams, W. J., & Bacon, S. (2016). Wind-driven mixing at intermediate depths in an ice-free Arctic Ocean. Geophysical Research Letters, 43, 9749–9756. https://doi.org/10.1002/2016GL070454

Reviewer #2 (Remarks to the Author):

Dear editor and authors,

This novel analysis uses unsupervised machine learning classification to identify distinct physical regimes of the Arctic surface momentum balance. Many of the conclusions are important and point to particular combinations of underlying mechanisms.

Thank you for this positive assessment of the novelty and potential importance of our work. We have carefully revised the manuscript to address the concerns raised below, particularly regarding the physical interpretation of the residual term and the robustness of the analysis.

The authors highlight the potential role of "internal stress divergence" of sea-ice as a contributor to the surface momentum balance. This may very well be the case in the real world. However, ECCOv4 is not the appropriate tool to identify the role of "internal stress divergence" of sea-ice, as ECCOv4 does not incorporate that in the momentum transfer between the ice and the ocean. I think the authors should therefore be more cautious in describing their important "residual" momentum component. In fact, I think an updated version of the manuscript should carefully consider the potential contributions to this residual component in greater detail. Because of the importance of this issue, I recommend Major Revisions.

Thank you for raising this issue. We agree that the original interpretation overstated the role of internal ice stress divergence within the ECCO framework. We have revised the manuscript to clarify that the residual term primarily reflects unresolved or parameterized processes in ECCO, rather than explicitly resolved internal ice stress dynamics (e.g., **Lines 340-364**). This revised interpretation is now consistent with the ECCO model formulation.

Major comments:

Lines 259-260: You list "unresolved processes such as internal stress divergence, small-scale deformation, and variable ice-ocean drag" - Actually, ECCOv4 does not use internal stress divergence and deformation of sea-ice when calculating the ice-ocean drag. ECCOv4 uses the Campin et al. (2008) formulation of ice-ocean drag rather than the Hibler and Bryan (1987) internal stress model.

So out of your list above, only the high-frequency ice-ocean covariability actually contributes to the residual term in ECCO. You should correct your statement.

Thank you for this important clarification. We agree that the original manuscript overstated the role of internal ice stress divergence within the ECCO framework. As

correctly noted, ECCOv4 does not explicitly include internal ice stress divergence in the formulation of ice–ocean momentum transfer.

We have therefore substantially revised the interpretation of the residual term throughout the manuscript. In the revised version, the residual is described more cautiously as representing unresolved or parameterized processes within ECCO, including high-frequency ice–ocean coupling variability, temporal averaging effects, and model–observation mismatch, rather than explicitly resolved internal ice stress dynamics (**Lines 350-364**).

We have also clarified the limitations of the ECCO framework in this context and removed statements that could be interpreted as attributing the residual directly to internal ice stress divergence. This revision ensures that the interpretation remains physically consistent with the model formulation.

Lines 290-292: "All non-residual momentum contributors -- surface wind stress... -- are directly observed or derived from satellite measurements, grounding the identified regimes in quantities that are routinely monitored..." - Please comment whether surface wind stress is accurately estimated in zones of large sea-ice concentration.

Thank you for this important point. We agree that surface wind stress is not directly observed with high accuracy in regions of high sea-ice concentration, where it is typically derived from atmospheric reanalyses and parameterized air–ice–ocean drag formulations.

We have revised the manuscript to clarify that the non-residual components are constrained by satellite observations to varying degrees. In particular, we now explicitly distinguish between directly observed quantities (e.g., sea-ice drift and sea surface height) and derived quantities (e.g., wind stress), and acknowledge the increased uncertainty in ice-covered regions (**Lines 350-364**).

Minor comments:

Figure 1 caption, lines 68-73: specify the sign convention, e.g., "negative downward"

We have revised the figure captions to explicitly state the sign convention used (**Lines 89-91**).

Lines 89-91: “Positive values correspond to upward Ekman transport (upwelling), while negative values indicate downward Ekman pumping (downwelling).”

Lines 111-111: "with both terms acting in the same sense within each regime" -

express more clearly the conclusion that the two terms are of the same sign.

We have revised this statement to explicitly indicate that the two terms share the same sign within each regime.

Lines 129-131: "Clusters C3 and C4 are characterized as sea-ice–geostrophic cooperative regimes, in which the ice and geostrophic contributions act in the same direction, with C3 and C4 differing primarily by a reversal in sign."

Figure 3 caption, lines 142-149: specify the sign convention, e.g., "positive upward"

We have revised this statement to explicitly state the sign convention used (**Lines 166-167**).

Figure 3, panels a and b, legend: it is a bit hard to distinguish the colors indicating C1 and C5, C2 and C6. Could you use markers of different shapes to distinguish among them?

We have revised the figure to improve visual clarity by changing the marker for each cluster.

Figure S1 caption, Line 43 in the Supplementary Information: "from satellite data" - specify what satellite data. What is the data source?

We have revised the Supplementary Information to explicitly specify the satellite products used, including their data sources and references.

Additional references:

J.-M. Campin, J. Marshall, and D. Ferreira. Sea ice–ocean coupling using a rescaled vertical coordinate z*. Ocean Modelling, 24(1–2):1–14, 2008. doi:10.1016/j.ocemod.2008.05.005.

W. D. Hibler, III and K. Bryan. A diagnostic ice-ocean model. J. Phys. Oceanogr., 17(7):987–1015, 1987. DOI: https://doi.org/10.1175/1520-0485(1987)017<0987:ADIM>2.0.CO;2

Reviewer #3 (Remarks to the Author):

Review of "Regime Transitions in Arctic Surface Momentum Balance Reveal Persistent and Predictable Dynamical States" by Chao Liu

This manuscript explores the relative contribution of decomposed Ekman pumping components on Arctic surface momentum balance using the ECCO reanalysis product and Gaussian Mixture Modelling to identify distinct Ekman pumping regimes around the Arctic. The study adds to a long line of work studying ocean dynamics using unsupervised clustering methods (e.g., Sun et al. 2020, Jones et al., 2019). However, while much past work has focussed on clustering the ocean state using thermohaline properties, these authors cluster specifically on Ekman pumping components, enabling a direct connection between the GMM components and Ekman pumping contributors.

Thank you for this clear and concise summary of our work. We appreciate the recognition that while unsupervised clustering is an established tool in oceanography, our objective was to shift the focus from the ocean state to the dynamic forcing. By clustering on the contributors to the momentum balance, we believe this manuscript provides a clearer diagnostic of how atmospheric and ice-ocean stresses drive Arctic circulation.

The work is well written, with an easy-to-follow method and clear results. However, given the volume of past GMM work, I was expecting some more details to the clustering method used. In addition, given the sensitivity of GMM to the input data used, I was unsure about the applicability of the results to a broader set of observational or modelling works. I recommend publication of this work after substantial rework of the manuscript based on the following points:

1) More clarity on the GMM method
a) The authors do not make a convincing case for why GMM is the most appropriate clustering method in this work. While I agree that deterministic methods like K-means are not appropriate given the spatiotemporal mixing and variability between clusters, I am not sure fitting the w components to Gaussians is more appropriate. Gaussians can be a better choice for distributions that have approximately Gaussian spreads, but it is unclear if the underlying training data here is approximately Gaussian. I suggest the authors provide a step-by-step illustration of the underlying distribution, and the fitted Gaussians, to reassure the reader that GMM is an appropriate method for the underlying training data.

We agree that the suitability of GMM depends on whether the data can be reasonably approximated as a mixture of Gaussian-distributed components.

In this framework, GMM does not assume that the full dataset follows a single Gaussian distribution. Rather, it represents the data as a superposition of multiple Gaussian components, each approximating a locally coherent cluster in feature space.

To support this assumption, we have added a new supplementary figure (**Fig. S6**) showing (a) the marginal distributions of each feature in an example cluster (C7), and (b) representative two-dimensional projections with overlaid Gaussian contours from the fitted GMM (sea-ice against geostrophic). These diagnostics demonstrate that, although the global distributions are not strictly Gaussian, the data exhibit locally coherent structures that are well approximated by Gaussian components. Noting in panel b, some of the clusters largely overlays (e.g., C7 & C1) which is consistent with Figure 2 and more importantly, it is because the clusters also exist and are measured/determined by the other 2 dimensions.

We have clarified this point in the Methods section and emphasize that GMM is used here as a probabilistic clustering tool to capture regime structure, rather than implying that the underlying physical processes are themselves Gaussian (**Lines 432-433**).

b) Similarly, I was unsure why K=7 clusters ended up being used as the optimal number of clusters. I understand that the AIC and BIC begin to change minimally for more than 7 clusters, which can provide a statistical basis for this choice. However, given your end-use is to understand ocean dynamics and Ekman pumping regimes, I feel that a somewhat dynamical explanation may be more convincing. For example, if you could show that, when some seasons are omitted from the GMM training, the ability of GMM to represent the key clusters is unaffected for K>7, that would be interesting and convincing. Or, if you could show that the PDF in space for each cluster (i.e., the probability obtained from GMM) begins to sharpen at K>7, then you could say that you have minimised the presence of "mixed" boundaries between clusters.

Thank you for the suggestion. We agree that, beyond statistical criteria such as AIC/BIC, it is important to demonstrate that the chosen number of clusters yields physically meaningful and well-separated regimes.

To address this, we evaluated the clustering "sharpness" as a function of the number of clusters K, defined as the mean maximum posterior probability across all samples (**Fig. S7a**). We find that assignment confidence increases rapidly and reaches a plateau for K = 5 to K = 7, indicating that the dominant regimes are already well separated in feature space. For larger values (K > 7), the mean confidence decreases (e.g., from ~0.98 at K = 7 to ~0.92 at K = 8), suggesting that additional clusters primarily subdivide existing regimes and introduce less distinct, more weakly separated states.

We further examine the distribution of posterior assignment probabilities for the selected solution (K=7; **Fig. S7b**), which is strongly skewed toward unity. This indicates that the vast majority of samples are assigned with high confidence, and that mixed-regime states (i.e., samples with comparable probabilities across clusters) are rare.

Together, these results demonstrate that the clustering is both robust and well constrained, and support the selection of K=7 as a balance between statistical criteria and physical interpretability, without over-partitioning the feature space.

c) GMM is typically sensitive to an initial "random seed" – i.e., where the initial Gaussians are generated in phase space before iteratively moving to the final distribution/cluster location. This sensitivity should be explored in the work. If the random seed is modified, what do the distributions look like? Do they change a lot? What about the probability associated with the clusters?

Thank you for raising this important point regarding the sensitivity of GMM to initialization. To mitigate this effect, all baseline fits were performed using multiple replicates (Replicates = 5), with the solution corresponding to the maximum likelihood retained.

To explicitly quantify robustness, we additionally performed 10 independent GMM realizations using different random seeds. We evaluate the resulting variability in the learned cluster structure in parameter space (i.e., cluster centroids). As shown in **Fig. S8a**, the mean standard deviation of cluster centroids across runs is small (order 10^{-1} in feature space), with the lowest variability observed in the residual-related component. This indicates that the identified regimes correspond to stable attractors in feature space rather than being sensitive to initialization noise.

We therefore conclude that the clustering solution is robust with respect to random initialization.

d) Figures 2 and 3: The assignment of clusters via GMM is determined purely by the fact that p>0.5 for a given cluster. Hence, the assessments in Fig 2 and 3 are somewhat misleading, as vast proportions of the clusters could have relatively mixed probabilities, e.g. 0.5
0.9, for example. This will show how much influence "mixed" regions have on these diagnostics (like ocean area ratio).

Thank you for raising this important point.

To assess this, we explicitly examined the distribution of posterior assignment probabilities (**Fig. S7**). The results show that the vast majority of samples are assigned with high confidence, with maximum posterior probabilities typically exceeding 0.9

and a distribution strongly skewed toward unity. This indicates that mixed-regime states (e.g., samples with comparable probabilities across multiple clusters) are rare.

We further verified that samples with lower confidence (e.g., $p < 0.7$ as shown in Figure 2a-d) are confined primarily to spatial boundary regions between regimes and constitute only a small fraction of the total area. As such, their influence on aggregated diagnostics such as cluster area ratios is minimal.

Therefore, while we agree that probabilistic mixing can in principle affect cluster-based interpretations, our results demonstrate that this effect is negligible in the present analysis due to the strong separation of regimes in feature space.

2) Applicability of method to alternative data sources
I was unsure of how robust this GMM method would be to different data sources. For example, if the GMM is highly sensitive to the nature of the input data, then these results may only hold true for ECCO and break down for other models or observational datasets. This would substantially weaken the conclusions, in my view. I encourage the authors to consider some alternative approaches.
Some options include:
a) Remove some training data randomly (spatially or temporally) and evaluate the robustness of the GMM results to this reduced training dataset.
b) Subsample training data to align with available observations and see how robust the GMM clusters are.
c) Remove future years or seasons and see if the trained GMM can fit well over these years or seasons (to assess the impact of seasonal biases in observations on results).
d) Assess other reanalysis or modelling datasets to determine the robustness of the results of different ocean-sea ice physics and forcing fields.

Thank you for this important comment regarding the robustness of the clustering framework to variations in the training dataset.

Because the Gaussian Mixture Model is applied in feature space (Ekman pumping components) and does not explicitly use spatial or temporal coordinates as inputs, we assess robustness by perturbing the input dataset through random subsampling prior to model fitting (**Lines 429-430**).

Specifically, we performed 10 independent experiments in which 20% of the input samples were randomly withheld, and the GMM was refitted for each realization. The resulting variability in cluster centroids is shown in **Fig. S8(b)**. We find that centroid variations remain small across realizations, indicating that the identified regimes are not strongly dependent on the specific sampling of the ECCO dataset.

Taken together, these results demonstrate that the clustering solution is stable under moderate perturbations of the training dataset. We agree that applying the framework

to alternative reanalysis or observational products would provide an additional and valuable test of generality, and we now explicitly highlight this as a direction for future work in the revised manuscript.

Minor Comments:

- How does the author compute each Ekman pumping contribution for each one of the clusters (fig 2 g).

In fig. 2g (and for GMM clustering), the Ekman pumping contributions are represented by the normalized ratios (γ, see Eq. 2). After obtaining the clusters, for each regime, we calculate the area-weighted mean of each Ekman pumping component (wind, sea-ice, geostrophic, and residual) using the grid cell area as weights.

This clarification has been added to **Lines 112-117**.

- The author talks about “a critical link between atmospheric forcing and the vertical redistribution of heat and freshwater, offering a roadmap for predicting how weakened ice cover will alter arctic-wide mixing and stratification” in the abstract. However, I was unable to find any mention of vertical distributions or stratification again in the discussion. The only mention was a brief discussion around the mixed layer.

We agree that the original wording in the abstract overstated the extent to which vertical redistribution and stratification are directly quantified in this study.

In the revised manuscript, we have moderated these statements to clarify that our analysis focuses on surface momentum balance and Ekman-driven processes, which are known to influence vertical exchange, but do not explicitly resolve stratification or subsurface structure.

We retain a forward-looking perspective by noting that the identified surface regimes provide a useful framework for understanding potential impacts on vertical exchange, while avoiding direct claims that are not explicitly demonstrated in the present analysis (**Lines 366–378**).

Minor Changes
- line 107 areal -> area
- fig 1 (f) -> (d).

Thanks for catching the errors. They have been revised accordingly.

Reviewer #1:

I appreciate the authors for their clarification and multiple modifications. The limitation of the study is now explicit presented. However, I was not clear enough, not just the temporal coherence and variability of ECCOv4r4 is different with the observational based method, but also their spatial patterns (in terms of your regime structure). This bias should also be mentioned.
In the abstract, you concluded “Spatially, we observe in the Beaufort and other marginal seas an increasing frequency of seasonal transitions from stable geostrophic-controlled states toward wind-driven downwelling.” I think this is related to lines 271-272 and lines 280-290 (Figures 4a-c), what I see is an increasing frequency of cluster 5 (C5), and this cluster reflects “ice drift induces Ekman upwelling, which is partially compensated by geostrophic downwelling” (in lines 141-142). This does not make sense to your claim that “increase seasonal transitions from stable geostrophic-controlled states toward wind-driven downwelling”. The wind-driven downwelling regime corresponds to cluster 2 (C2), which exhibits persistently zero values throughout the study periods (Figure 4c)!

Thank you for raising this important clarification. Upon re-examining the regime definitions and temporal evolution shown in Figures 4a-c, we agree that our previous wording was overly broad and did not sufficiently distinguish the differing regional behaviors between the Beaufort Sea and the Nordic/Eurasian sector.

In the original manuscript, the phrase “transition from stable geostrophic-controlled states toward wind-driven downwelling” was intended to summarize the increasing prevalence of dynamically active regimes across Arctic marginal seas. However, as correctly noted by the reviewer, the dominant evolution differs substantially between the two regions.

For the Beaufort Sea (Figures 4a), the primary increase is associated with Cluster 5 & 6 (C5 & C6), as the 5th & 6th columns are mostly marked with higher probability. According to the regime definitions established in Figure 2g, C5 represents a partially compensated ice–geostrophic regime in which ice-drift-induced Ekman upwelling is balanced by geostrophic downwelling. Therefore, the Beaufort evolution does not indicate a systematic shift toward a purely wind-driven downwelling state. Instead, it reflects a transition away from highly persistent geostrophic-balanced regimes (primarily C3-C4) toward more dynamically active states with stronger ice-drift and wind influence, while geostrophic compensation remains important.

In contrast, the Nordic/Eurasian sector (Figures 4b & 4c) exhibits increasing occurrence and persistence of Cluster 1 (C1) and Cluster 2 (C2), i.e., the 1st & 2nd columns are mostly marked with higher probability. which corresponds more directly to a wind-driven downwelling regime characterized by strong positive wind contribution with relatively weak compensating terms. In this region, the

interpretation of increasing wind-driven influence is therefore more appropriate and consistent with the diagnosed cluster evolution.

We agree that the previous wording in the Abstract did not adequately separate these distinct regional transitions and could therefore be misleading. We have revised the corresponding statements throughout the manuscript to distinguish the Beaufort and Nordic/Eurasian behaviors more explicitly. The revised text now emphasizes that the Beaufort Sea primarily shifts toward partially compensated ice–geostrophic states, whereas the Nordic/Eurasian sector shows increasing prevalence of wind-driven downwelling regimes. ((Lines 22-25)

We have also added clarification that uncertainties in ECCOv4r4 relative to observation-based estimates may influence not only the temporal variability of the diagnosed momentum components, but also the spatial organization and relative occurrence of the identified dynamical regimes. (Lines 399-403)

We believe this revised interpretation is more consistent with the diagnosed cluster structure and better reflects the physical meaning of the identified regime transitions.

Reviewer #1:

This is my third-round review of Liu and Liang 2026. My evaluation is that their study brings new insight into the changing dynamic regime of Ekman pumping in the Arctic Ocean. But I still don't follow some of the arguments that were presented in the paper.

Thank you for the careful re-evaluation and for pointing out the ambiguity in our interpretation of the transition and persistence diagnostics. We agree that our previous wording did not sufficiently distinguish between transition probability, persistence probability, and temporal changes in regime occurrence. In this round of revision, we tried to remove the ambiguity and make the arguments easier to follow.

For their response "For the Beaufort Sea (Figures 4a), the primary increase is associated with Cluster 5 & 6 (C5 & C6), as the 5th & 6th columns are mostly marked with higher probability.", I only see an increase in Cluster 5 in Figure 4c. The Cluster 4 shows an increase until 2006-2007, and then it gradually decreased (Figure 4c). The mentioned Figure 4a only shows the probability of transition from one cluster to another cluster, but it does not imply the trend (increase).

As you correctly noted, Figure 4a does not itself demonstrate a temporal increase in cluster occurrence. It is constructed by counting area-weighted transitions of each grid cell between cluster regimes that change state from one year to the next. Figure 4a therefore represents the mean transition probability matrix over the full analysis period (1992-2017) and diagnoses the preferential pathways of regime transitions, i.e., the off-diagonal elements (P_{ij}). In this panel/framework, the relatively elevated probabilities in the C5 and C6 columns indicate that transitions from multiple regimes preferentially terminate in these dynamically active states within the Beaufort Sea sector. In other words, C5 and C6 act as comparatively favored transition states in the mean regime dynamics.

By contrast, Figures 4b and 4c quantify persistence behavior through time. Figure 4b shows the fraction of area remaining within the same regime between consecutive years, while Figure 4c shows the persistence probability of each individual cluster (i.e., the diagonal elements of the transition matrix in Figure 4a, P_{ii}).

For instance, the large diagonal value for C6 in Figure 4a indicates that, on average, grid cells assigned to C6 are more likely to remain in the same regime from one year to the next, implying relatively high persistence. Figure 4c further shows how this persistence probability is between 0.8-0.9 over the time, demonstrating that C6 remains consistently stable throughout most of the record. These diagnostics therefore describe temporal stability rather than transition preference.

To address this, we have revised lines 272 to 314 to explicitly state that Figure 4a represents the mean transition probability matrix aggregated over the entire analysis period and is used to diagnose preferred pathways of regime change (off-diagonal elements, e.g., lines 272-277), whereas Figures 4b–i quantify regime persistence and its temporal evolution (diagonal elements, e.g., lines 280-285).

In another response, “In contrast, the Nordic/Eurasian sector (Figures 4b & 4c) exhibits increasing occurrence and persistence of Cluster 1 (C1) and Cluster 2 (C2)”, the Nordic/Eurasian sector does not refer to your Figures 4b & 4c! And I don’t see Cluster 1 and Cluster 2 exhibit increasing occurrence, what I see from Figures 4f & 4i are persistence of Cluster 1 and Cluster 2 rather than increasing. How do you conclude that the Nordic/Eurasian sector exhibits an increasing wind-driven downwelling regime?

Regarding this point, we confirm that the reference to Figures 4b&4c in our previous response was incorrect; the correct panels are Figures 4d and 4g, which describe transition probabilities in the Nordic/Eurasian sector.

The quantities shown in Figures 4f and 4i represent regime persistence (diagonal elements of the transition matrix) and its temporal variability, rather than direct changes in regime occurrence. Therefore, they should not be interpreted as a monotonic increase in the frequency of Cluster 1 or Cluster 2.

What the results do indicate is a relative strengthening of regime stability for C1 and C2 toward the latter part of the record in terms of persistence, rather than an increase in their spatial occurrence or occupancy.

We have revised the manuscript accordingly to avoid implying that the transition matrix itself demonstrates temporal increases in cluster occurrence. We have also removed any implication of a trend in occurrence and now restrict the interpretation to persistence and transition structure only (Lines 272-314).

The revised conclusion in the abstract, “Spatially, the Beaufort Sea shifts from persistent geostrophic-balanced states toward more dynamically active ice–geostrophic regimes, whereas the Nordic/Eurasian marginal seas exhibit increasing prevalence of wind-driven downwelling states.” was not even mentioned in the main text.

We apologize for not being careful enough about this. The abstract statement has now been revised to:

“Spatially, the Beaufort Sea is characterized by frequent transitions and enhanced persistence of ice–geostrophic coupled regimes, whereas the Nordic and Eurasian marginal seas exhibit greater variability in regime stability over time.” (Lines 22-26)

Thank you again for your help in improving the quality of this paper. We hope these revisions addressed the issues you raised and provide a more careful and internally consistent interpretation of the diagnosed regime behavior.
